# Intersectin and endophilin condensates prime synaptic vesicles for release site replenishment

**DOI:** 10.1038/s41593-025-02002-4

**Published:** 2025-07-08

**Authors:** Tyler H. Ogunmowo, Christian Hoffmann, Chintan Patel, Renee Pepper, Han Wang, Sindhuja Gowrisankaran, Johanna Idel, Annie Ho, Sumana Raychaudhuri, Brady J. Maher, Benjamin H. Cooper, Ira Milosevic, Dragomir Milovanovic, Shigeki Watanabe

**Affiliations:** 1 Department of Cell Biology, Johns Hopkins University School of Medicine, Baltimore, MD USA; 2 Laboratory of Molecular Neuroscience, German Center for Neurodegenerative Diseases (DZNE), Berlin, Germany; 3 Solomon H. Snyder Department of Neuroscience, Johns Hopkins University School of Medicine, Baltimore, MD USA; 4 Lieber Institute for Brain Development, Johns Hopkins University School of Medicine, Baltimore, MD USA; 5 European Neuroscience Institute (ENI), Göttingen, Germany; 6 Department of Psychiatry and Behavioral Sciences, Johns Hopkins University School of Medicine, Baltimore, MD USA; 7 Department of Molecular Neurobiology, Max Planck Institute for Multidisciplinary Sciences, Göttingen, Germany; 8 Centre for Human Genetics, Nuffield Department of Medicine, NIHR Oxford Biomedical Research Centre, University of Oxford, Oxford, UK; 9 Multidisciplinary Institute of Ageing, Centre for Innovative Biomedicine and Biotechnology, University of Coimbra, Coimbra, Portugal; 10 Institute of Biochemistry & Einstein Center for Neuroscience, Charité-Universitätsmedizin Berlin, Corporate Member of Freie Universität Berlin, Humboldt-Universität Berlin and Berlin Institute of Health, Berlin, Germany; 11 German Center for Neurodegenerative Diseases (DZNE), Bonn, Germany

**Keywords:** Synaptic vesicle exocytosis, Exocytosis, Cellular neuroscience, Vesicle trafficking

## Abstract

Following synaptic vesicle fusion, vacated release sites are replenished immediately by new vesicles for subsequent neurotransmission. These replacement vesicles are assumed to be located near release sites and used by chance. Here we find in mouse hippocampal excitatory synapses that replacement vesicles are clustered near the active zone where release sites reside by intersectin-1. Specifically, intersectin-1 forms dynamic molecular condensates with endophilin A1 and sequesters vesicles around this region. In the absence of intersectin-1, fewer vesicles cluster within 20 nm of the plasma membrane, and consequently vacated sites cannot be replenished rapidly, leading to synaptic depression. Mutations in intersectin-1 that disrupt endophilin A1 binding result in similar phenotypes. In the absence of endophilin A1, intersectin-1 is mislocalized, and this replacement pool of vesicles cannot be accessed, suggesting that endophilin A1 is needed to mobilize these vesicles. Thus, our work describes the replacement zone within a synapse, where replacement vesicles are stored for replenishment of the release site.

## Abstract (web summary)

Time-resolved electron microscopy reveals that intersectin-1 and endophilin A1 condensates hold replacement synaptic vesicles close to release sites. Without this, replacement vesicles are unavailable for immediate use, causing synaptic depression in response to stimulation trains.

## Main

At chemical synapses, signaling is mediated by the calcium-dependent exocytosis of synaptic vesicles^1,2^. In resting synapses, synaptic vesicles can dock at release sites within the active zone, where release machinery is concentrated^3–10^. At any given synapse, there exist far fewer release sites than vesicles, and at each release site, only one vesicle can dock^11–13^. This limitation sets an upper boundary for the number of vesicles that are release-ready at any given time. Thus, for a synapse to resist depression of synaptic transmission or to enhance synaptic signaling, the vacated sites must be actively replenished. As such, this replenishment is rate-limiting for continued neurotransmitter release.

Traditionally, the replenishment of release sites was thought to be slow, requiring ~2 to 10 s^14–17^. However, synapses are capable of resisting depression of neurotransmitter release during high-frequency stimulation and even enhancing transmission within tens of milliseconds^12,18,19^, suggesting that replenishment can occur rapidly. In line with this, electrophysiological recordings paired with mathematical modeling suggest that there exists a pool of vesicles that respond to docked vesicle depletion and supply new vesicles from so-called ‘replacement sites’ to release sites after fusion^20^. Furthermore, ultrastructural analysis suggests that vesicles can transiently dock to resist depression and enhance synaptic strength in a calcium-dependent fashion^7,21^, a process that may reflect the transition of a replacement vesicle to a docked vesicle. Thus, release sites can be replenished on a millisecond time scale after fusion events^20,22–25^.

There are several functional pools of synaptic vesicles, including the readily releasable pool (RRP), reserve pool, recycling pool and the multiple synapse-spanning superpool^11,26–29^. Recent studies suggest that some of these pools are separated into distinct physical domains within a synapse by a molecular condensation process^30^. For example, the RRP of vesicles may be organized by the condensation of active zone proteins^31–34^. Additionally, a few synaptic vesicles are thought to tether to these active zone phases^35,36^. Similarly, the reserve pool of synaptic vesicles is separated by the multivalent interactions of the synaptic vesicle binding protein synapsin 1 (Syn1) on vesicle membranes^37–39^. Thus, synaptic vesicles are physically organized through molecular condensation, which engenders specific functional roles at synapses.

The reserve pool is suggested to also contain proteins like intersectin-1 (Itsn1) and endophilin A1 (EndoA1)^38,40–43^. However, Itsn1 and EndoA1’s function may lie outside of the reserve pool. Non-neuronal secretory cells display Itsn1 enrichment at sites of granule secretion, and its knockdown perturbs hormone release during sustained activity^44^. More recently, work in neuroendocrine adrenal chromaffin cells shows that Itsn1 together with EndoA1 mediates granule replenishment during stimulation^45^. At the calyx of Held, fast vesicle replenishment seems to be abolished in Itsn1 knockout (KO) neurons^46^. Furthermore, the absence of either Itsn1 or EndoA1 in mouse hippocampal synapses leads to synaptic depression during a train stimulus^47,48^. These data suggest that Itsn1 and EndoA1 may contribute to activity-dependent replenishment of release sites.

Here we demonstrate that Itsn1–EndoA1 form condensates and mediate the maintenance of the replacement vesicle pool between the active zone and the reserve pool in the so-called replacement zone. Specifically, Itsn1 and EndoA1 are located between the active zone and the Syn1-positive reserve vesicle cluster. In neurons lacking Itsn1 or the functional interaction of Itsn1 with EndoA1, the number of vesicles within 20 nm of the active zone is substantially reduced. Without EndoA1, replacement vesicles are intact, yet not accessible for docking. Consequently, these vesicles are mislocalized or nonfunctional; thus, the replenishment of release sites is slowed, which leads to accelerated depression of synaptic transmission.

## Results

### Itsn1 condenses with synaptic proteins and vesicles

**Fig. 1:**
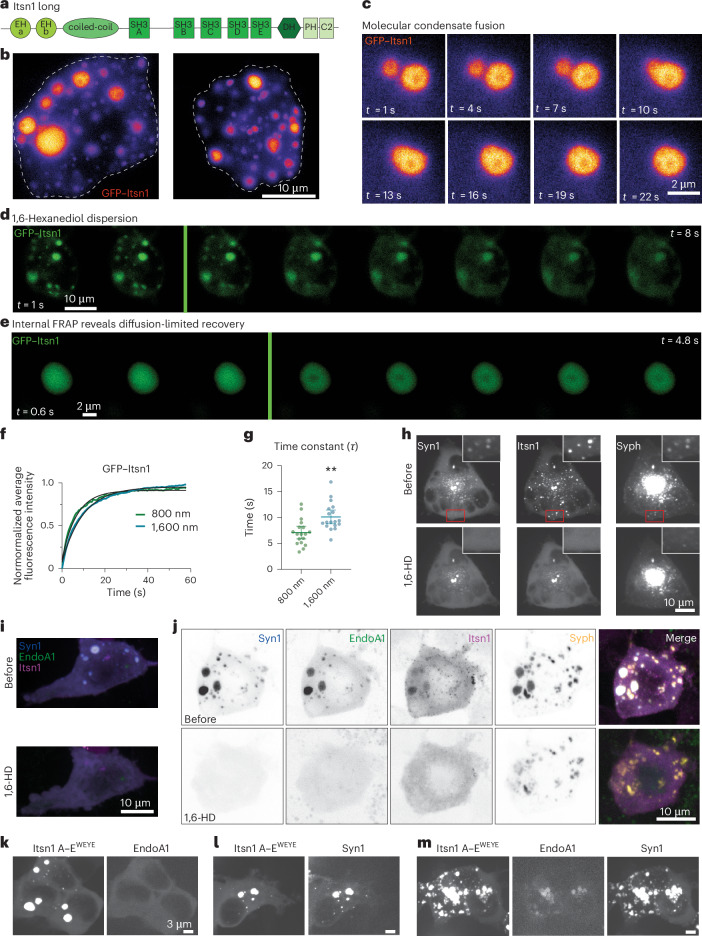
Itsn1 forms condensates containing presynaptic proteins and structures. **a**, The protein domain structure of Itsn1. **b**, Two HEK293T cells expressing GFP–Itsn1 FL. Scale bar, 10 µm. **c**, Example live-HEK293T cell images showing GFP–Itsn1 FL condensates undergoing a fusion event. Times after the initiation of image acquisitions are indicated. Scale bar, 2 µm. **d**, Example live-cell fluorescence micrographs showing GFP–Itsn1 signals over 8 s with the addition of 4% 1,6-HD after 2 s (green line). Scale bar, 10 µm. **e**, Fluorescence recovery of GFP–Itsn1 signals when signals within the condensate were photobleached (green line) with a diameter of 800 nm. Each frame represents 0.6 s. Scale bar, 2 µm. **f**, Plot showing FRAP with a bleaching spot of the indicated diameters in HEK293T cells expressing GFP–Itsn1. **g**, Plot showing time constant Tau of fluorescence recovery in **f**. Bars are the mean; error bars are s.e.m. Student’s *t*-test. **P = 0.0013. **h**, Top, HEK293 cells co-expressing mCherry–Syn1, GFP–Itsn1 FL and synaptophysin-emiRFP (red fluorescent protein) 670 (Syph). Bottom, cells in top after 3% 1,6-HD treatment. Scale bar, 10 µm. **i**, Top, HEK293 cells co-expressing BFP–Syn1, mCerulean–EndoA1 and HaloTag–JF549-Itsn1 FL. Bottom, cells after 3% 1,6-HD treatment. Scale bars, 10 µm. **j**, Top, HEK293 cells co-expressing BFP–Syn1, mCerulean–EndoA1, HALO (JF549)–Itsn1 FL and Syph–emiRFP670. Each individual channel is separated and then merged. Bottom, cells in top after 3% 1,6-HD treatment. Scale bars, 10 µm. **k**–**m**, HEK293 cells co-expressing mutant mCherry–Itsn1 A–E (Itsn1 W949E and Y965E; Itsn1 A–E^WEYE^) and mCerulean–EndoA1 (**k**), mCherry–Itsn1 A–E^WEYE^ and BFP–Syn1 (**l**) and mCherry–Itsn1 A–E^WEYE^, mCerulean–EndoA1 and BFP–Syn1 (**m**). Scale bar, 3 µm. See Supplementary Table 1 for additional information. HD, hexanediol. Source data

**Extended Data Fig. 1:**
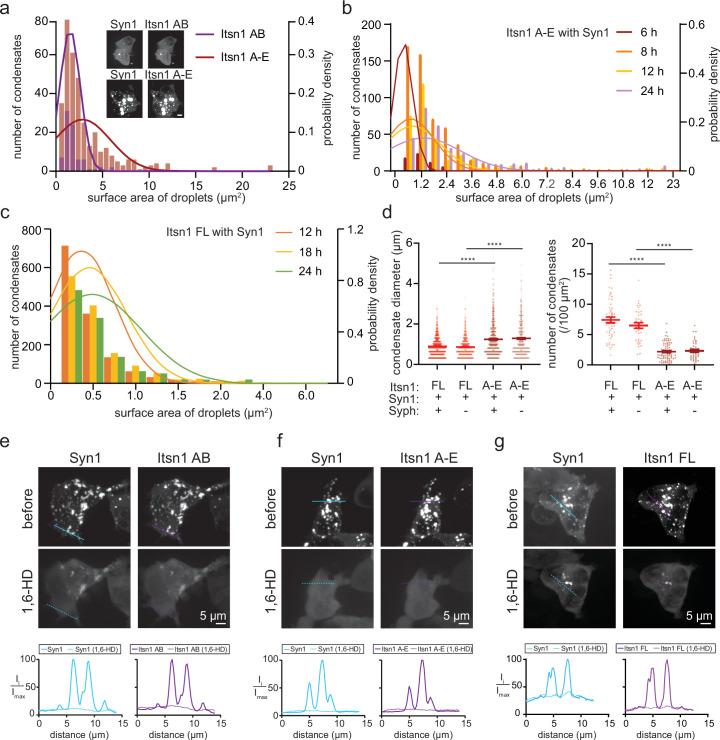
Itsn1 and Syn1 form reversible condensates. **a**, A bar graph representing the number of condensates formed by co-expression of Syn1 with either a concatemer of two (Itsn1 AB, purple) or five (Itsn1 A–E, red) Itsn1 Src-homology 3 (SH3) domains, overlaid with the line graph representing the frequency distribution. Inset, representative images of HEK293 cells co-transfected with GFP–Syn1 and mCherry–Itsn1 AB or mCherry–Itsn1 A–E, respectively. Scale bar, 5 µm. **b**, A bar graph representing the number of Itsn1 A–E condensates with Syn1 (co-expression of mCherry–Itsn1 A–E and GFP–Syn1) at the indicated time after transfection, overlaid with the line graph representing the frequency distribution. Note that the effective concentration increases with time after transfection. **c**, A bar graph representing the number of Itsn1 FL condensates with Syn1 (co-expression of GFP–Itsn1 FL and mCherry–Syn1) at the indicated time after transfection, overlaid with the line graph representing the frequency distribution. Note that the effective concentration increases with time after transfection. **d**, Left, plot showing the condensate diameter of Itsn1 FL (red)/A–E (dark red)-Syn1 condensates with or without co-expression of untagged Syph. Itsn1 FL/Syn1 are tagged as in **c**, Itsn1 A–E/Syn1 are tagged with mCherry and GFP, respectively. Bars are the mean; error bars are s.e.m. Kruskal–Wallis test, with Dunn’s multiple comparisons test. The condensates are analyzed from three independent transfections; ****p < 0.0001 was calculated when comparing Itsn1 FL–Syn1–Syph co-expressing cells to Itsn1 A–E-Syn1–Syph co-expressing cells, and ****p < 0.0001 was calculated when comparing Itsn1 FL–Syn1 co-expressing cells to Itsn1 A–E–Syn1 co-expressing cells. Right, plot showing the number of Itsn1 FL/A–E–Syn1 condensates with or without co-expression of untagged Syph. Bars are the mean; error bars are s.e.m. Kruskal–Wallis test, with Dunn’s multiple comparisons test. The condensates are analyzed from three independent transfections; ****p < 0.0001 was calculated when comparing Itsn1 FL–Syn1–Syph co-expressing cells to Itsn1 A–E-Syn1–Syph co-expressing cells, and ****p < 0.0001 was calculated when comparing Itsn1 FL–Syn1 co-expressing cells to Itsn1 A–E–Syn1 co-expressing cells. **e**, Top, representative images of HEK293 cells co-expressing GFP–Syn1 and mCherry–Itsn1 AB domains before and after application of 3% 1,6-hexanediol (1,6-HD). Bottom, plots showing fluorescence intensity of Syn1 (blue) and Itsn1 AB (purple) at the indicated line on the images before (solid line) and after (dotted line) the application of 1,6-hexanediol. Scale bar, 5 µm. **f**, Top, representative images of HEK293 cells co-expressing GFP–Syn1 and mCherry–Itsn1 A–E domains before and after application of 3% 1,6-hexanediol (1,6-HD). Bottom, plots showing fluorescence intensity of Syn1 (blue) and Itsn1 A–E (purple) at the indicated line on the images before (solid line) and after (dotted line) the application of 1,6-hexanediol. Scale bar, 5 µm. **g**, Top, representative images of HEK293 cells co-expressing GFP–Syn1 and HaloTag–JF549–Itsn1 FL domains before and after application of 3% 1,6-hexanediol (1,6-HD). Bottom, plots showing fluorescence intensity of Syn1 (blue) and Itsn1 FL (purple) at the indicated line on the images before (solid line) and after (dotted line) the application of 1,6-hexanediol. Scale bar, 5 µm. See Supplementary Table 1 for additional information. Source data

Itsn1 long is a neuronally enriched isoform of Itsn1 (Fig. 1a). Itsn1 has five Src-homology 3 (SH3) domains in tandem (namely, A–E; Fig. 1a), which enable interaction with a multitude of synaptic proteins, including Syn1 and EndoA1 (refs. ^37,38,41,43,49^). When EGFP (enhanced green fluorescent protein)–Itsn1 (hereafter, GFP–Itsn1) was overexpressed alone in HEK293T cells, Itsn1 readily formed large puncta (Fig. 1b) that exhibited the key properties of biomolecular condensates^50^—they readily fused with one another (Fig. 1c), were dispersed by the application of 1,6-hexanediol (Fig. 1d), an aliphatic alcohol that disrupts associative interactions in the case of condensates^51^, and promptly recovered fluorescence after photobleaching (Fig. 1e). In fact, fluorescence recovery after photobleaching (FRAP) measurements clearly indicate that the mobile fraction within Itsn1 condensates depends on the diameter of photobleaching (Fig. 1e–g), suggesting that molecules remain highly mobile within condensates^39,51^. Similar results were obtained from expressing full-length (FL, denoted hereafter for clarity) Itsn1 or concatemers of Itsn1 containing either only two (Itsn1 AB) or five (Itsn1 A–E) SH3 domains in the presence of co-expressed Syn1 (Extended Data Fig. 1a–c,e–g). Strikingly, Itsn1 FL and Itsn1 A–E condensates can contain synaptic vesicle-like organelle clusters (Fig. 1h and Extended Data Fig. 1d), which are generated in heterologous cells by co-expression of Syn1 and synaptophysin 1 (Syph)^42^ and retain 1,6-hexanediol sensitivity (Fig. 1h). Together, these data show that Itsn1 dynamically condenses in cells and can sequester synaptic vesicle-like organelles^37,42^.

### EndoA1 coalesces with Itsn1 condensates

**Extended Data Fig. 2:**
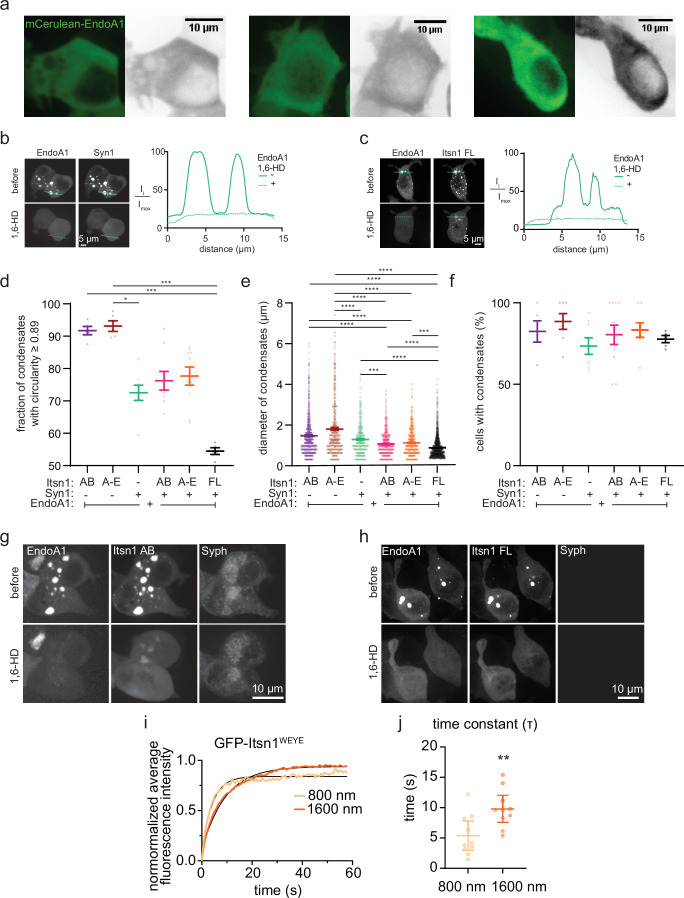
Itsn1 and EndoA1 can assemble into distinct condensates. **a**, Three example images of live HEK293 cells expressing mCerulean–Endo A1. Scale bar, 10 µm. **b**, Left, HEK293 cells co-expressing mCherry–Syn1 and mCerulean–EndoA1 before (top) and after application of 3% 1,6-hexanediol (bottom). Right, plot showing fluorescence intensity of EndoA1 (green) at the indicated line on the images before (solid line) and after (dotted line) the application of 1,6-hexanediol. Scale bar, 5 µm. **c**, Same as **b**, but with GFP–Itsn1 FL and BFP–EndoA1. **d**, Plot showing the fraction of condensates with high circularity (≥0.89 ratio of x and y diameter, where x is the smaller axis) of either Itsn1–EndoA1, Syn1–EndoA1 or Itsn1–EndoA1–Syn1 condensates. The concatemer of Itsn1 was either AB or A–E. Itsn1 AB/A–E are mCherry, Itsn1 FL is fused to HaloTag–JF549, EndoA1 is mCerulean-tagged and Syn1 is BFP-tagged. Bars are the mean; error bars are s.e.m. Kruskal–Wallis test, with Dunn’s multiple comparisons test. The condensates are analyzed from three independent transfections; *p = 0.0332 was calculated when comparing Itsn1 A–E–EndoA1 to Syn1–EndoA1, ***p = 0.0003 was calculated when comparing Itsn1 FL–Syn1–EndoA1 to Itsn1 AB–EndoA1, and ***p=0.0001 was calculated when comparing Itsn1 FL–Syn1–EndoA1 to Itsn1 A–E–EndoA1 or Itsn1 A–E–EndoA1. **e**, Plot showing the diameter of condensates containing either Itsn1–EndoA1, Syn1–EndoA1, or Itsn1–EndoA1–Syn1. Fusion tags are the same as **d**. The concatemer of Itsn1 was either AB or A–E. Bars are the mean; error bars are s.e.m. Kruskal–Wallis test, with Dunn’s multiple comparisons test. The condensates are analyzed from three independent transfections; ****p < 0.0001 was calculated when comparing Itsn1 AB–EndoA1 to either Itsn1 AB–Syn1–EndoA1, Itsn1 A–E–Syn1–EndoA1 or Itsn1 FL–Syn1–EndoA1. ****p < 0.0001 was calculated when comparing Itsn1 A–E–EndoA1 to either Syn1–EndoA1, Itsn1 AB–Syn1–EndoA1, Itsn1 A–E–Syn1–EndoA1 or Itsn1 FL–Syn1–EndoA1. ***p = 0.0008 was calculated when comparing Syn1–EndoA1 to Itsn1 AB–Syn1–EndoA1 and ****p < 0.0001 was calculated when comparing Syn1–EndoA1 to Itsn1 FL–Syn1–EndoA1. ****p < 0.001 was calculated when comparing Itsn1 FL–Syn1–EndoA1 to Itsn1 AB–Syn1–EndoA1 or Itsn1 A–E-Syn1–EndoA1. **f**, Plot showing the fraction of cells containing either Itsn1–EndoA1, Syn1–EndoA1 or Itsn1–EndoA1–Syn1 condensates. The concatemer of Itsn1 was either AB or A–E. Fusion tags are the same as in **d** and **e**. Bars are the mean; error bars are s.e.m. **g**, Top, HEK293 cells expressing mCerulean–EndoA1, mCherry–Itsn1 AB and Syph–emiRFP670. Bottom, cells in the top after treatment with 3% 1,6-hexanediol. Scale bar, 10 µm. **h**, Top, HEK293 cells expressing BFP–EndoA1, GFP–Itsn1 FL and Syph (untagged). Bottom, cells in top after treatment with 3% 1,6-hexanediol. Scale bar, 10 µm. **i**, Same as Fig.1f, but with GFP–Itsn1^WEYE^. **j**, Same as Fig. 1g, but with GFP–Itsn1^WEYE^. Bars are the mean; error bars are s.e.m. Two-sided Student’s *t* test. **p = 0.0069. See Supplementary Table 1 for additional information. Source data

Recently, EndoA1 was shown to both facilitate the phase separation of Syn1 and enter synaptic vesicle-like clusters through interaction with Syn1 (ref. ^43^). To test whether EndoA1 is also found in Itsn1 condensates, we expressed EndoA1 with Itsn1 FL, Itsn1 AB or Itsn1 A–E. Unlike Itsn1, EndoA1 expressed alone was largely diffuse within cells (Extended Data Fig. 2a). However, EndoA1 formed large, 1,6-hexanediol-sensitive condensates with Syn1 when co-expressed (Extended Data Fig. 2b). When co-expressing Itsn1 FL, EndoA1 and Syn1, 1,6-hexanediol-sensitive condensates also formed in cells (Fig. 1i). Condensates formed by differential combinations of Itsn1 FL, Itsn1 AB, Itsn1 AE, Syn1 and EndoA1 all displayed distinct circularities and sizes (Extended Data Fig. 2d,e), yet they formed within cells at the same frequency (Extended Data Fig. 2f), suggesting molecular composition changes condensate properties without affecting their formation likelihood, similar to other ectopically generated synaptic condensates^52^. Notably, Itsn1 FL condensates readily incorporated EndoA1 in the absence of Syn1 and retained 1,6-hexanediol sensitivity (Extended Data Fig. 2c), indicating their interaction independently of Syn1. When co-expressed with Syph, Itsn1 FL-EndoA1 condensates formed, and they were dispersed by 1,6-hexanediol (Extended Data Fig. 2g,h). However, unlike Itsn1 FL–Syn1 condensates (Fig. 1h), they did not contain Syph signal (Extended Data Fig. 2g,h), indicating that Syph can only enter Itsn1–EndoA1 condensates when it is on synaptic vesicle-like organelles whose formation requires Syn1 co-expression^42^.

These data suggest molecular and organelle selectivity by Itsn1–EndoA1 condensates. In line with this, when we expressed all four proteins, they formed condensates (Fig. 1j) that can be dispersed by 1,6-hexanediol (Fig. 1j), suggesting that these Itsn1 FL-EndoA1 cocondense with synaptic vesicle-like organelle clusters. Finally, this recruitment of EndoA1 to Itsn1 condensates required their specific interaction, an unconventional SH3–SH3 interaction^41^. When Itsn1 A–E’s SH3B domain was mutated (W949E/Y965E) to block EndoA1 interaction as reported previously^41^ (Itsn1 A–E^WEYE^), Itsn1 condensates completely lacked EndoA1 (Fig. 1k). This is likely due to the lack of direct biochemical interaction—GFP–Itsn1^WEYE^. Itsn1 FL containing the abovementioned point mutations condensates displayed FRAP recovery kinetics identical to FL wild-type (WT) GFP–Itsn1 (Extended Data Fig. 2i,j), suggesting the viscosity of these condensates is not changed by these mutations. Consistently, Itsn1 A–E^WEYE^ condensates incorporated Syn1 normally (Fig. 1l). Interestingly, if Syn1 and Itsn1 A–E^WEYE^ are cotransfected with EndoA1, EndoA1 is found within Itsn1–Syn1 condensates (Fig. 1m), supporting the notion that Syn1 condensates facilitate the accumulation of both Itsn1 and EndoA1, likely through Syn1’s own interactions with both proteins^38^. Together, our results suggest that Itsn1 can form dynamic assemblies on synaptic vesicle-like clusters, which can contain EndoA1, indicating that Itsn1 condensates can potentially regulate vesicle dynamics in concert with EndoA1.

### Itsn1 and EndoA1 colocalize on vesicles near active zones

**Fig. 2:**
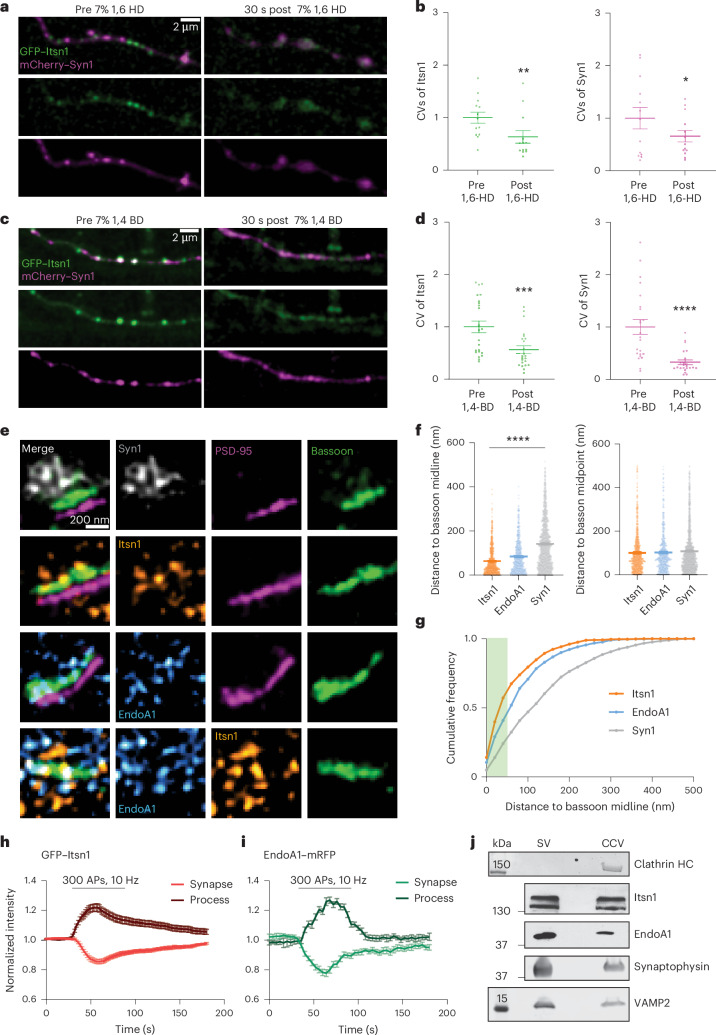
Itsn1 forms dynamic condensed structures that are active zone-adjacent and overlap EndoA1. **a**, Left, axon co-expressing GFP–Itsn1 and mCherry–Syn1 before the application of 7% 1,6-HD. Scale bar, 2 µm. Right, the same axon, but 30 s after the application of 7% 1,6-HD. **b**, CV of GFP–Itsn1 and mCherry–Syn1 in **a**. Bars are the mean; error bars are s.e.m. Two-sided Student’s *t*-test. *P = 0.0313 and **P = 0.0013. **c**, Left, axon co-expressing GFP–Itsn1 and mCherry–Syn1 before the application of 7% 1,4-BD. Scale bar, 2 µm. Right, the same axon, but 30 s after the application of 7% 1,6-BD. **d**, Same as **b**, CV of GFP–Itsn1 and mCherry–Syn1 in **c**. Bars are the mean; error bars are s.e.m. Two-sided Student’s *t*-test. ***P = 0.0001 and ****P < 0.0001. **e**, Three-color 2D STED representative images of side-view synapses. Each row is a different synapse stained for either Syn1, Itsn1 or EndoA1 along with the synaptic markers PSD-95 and/or Bassoon, with separate channels and merged channels displayed. Scale bar, 200 nm. **f**, Left, plot showing distances (nm) between either Itsn1, EndoA1 or Syn1 puncta to the Bassoon signal midline. Bars are the mean; error bars are s.e.m. Kruskal–Wallis test, with Dunn’s multiple comparisons test. ****P < 0.0001. Comparisons between Itsn1, EndoA1 and Syn1 to the Bassoon signal midline. Right, plot showing distances between either Itsn1, EndoA1 or Syn1 puncta to the midpoint of the Bassoon signal. Bars are the mean; error bars are s.e.m. **g**, Cumulative plot showing the distribution of Itsn1, EndoA1 and Syn1 puncta distances from the Bassoon midline. **h**, Plots showing GFP–Itsn1 fluorescence intensity at synaptic boutons or axonal processes. Traces shown are average values; error bars are s.e.m. **i**, Plots showing EndoA1–mRFP fluorescence intensity at synaptic boutons or axonal processes. Traces shown are average values; error bars are s.e.m. **j**, Different blots showing the indicated proteins on purified SV and CCV. Actual protein size marker or size indicator (in kDa) on the left-hand side. Fluorescence intensity in **h** and **i** was measured as depicted in Extended Data Fig. 3i and normalized to the baseline. See Supplementary Table 1 and source data for additional information. SV, synaptic vesicles; CCV, clathrin-coated vesicles; BD, butanediol. Source data

**Extended Data Fig. 3:**
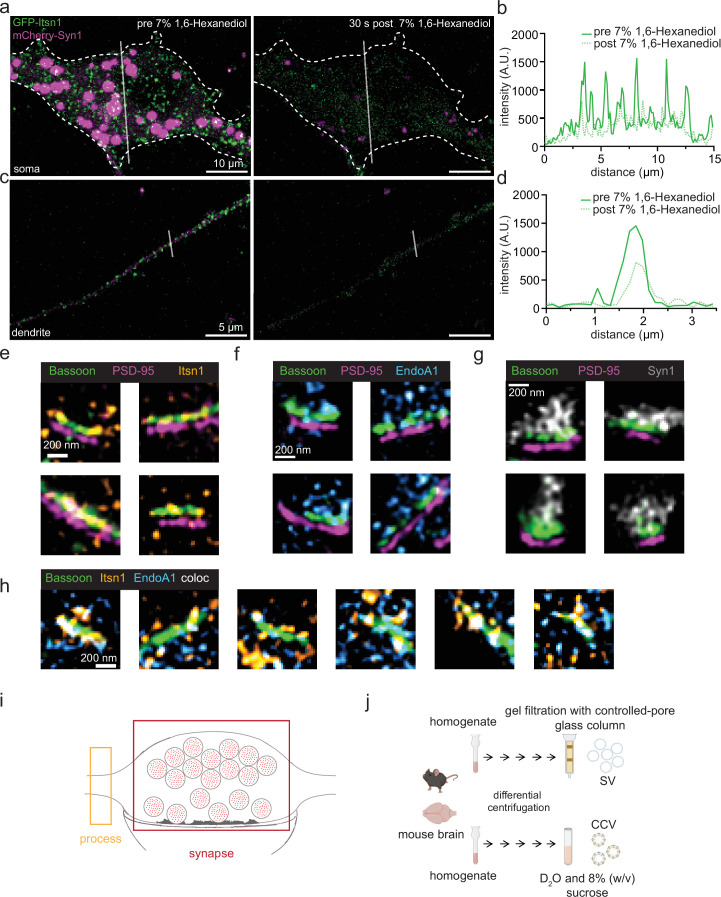
Itsn1 condensates localize near the active zone with EndoA1 on synaptic vesicles. **a**, Left, example micrograph of a neuronal soma expressing GFP–Itsn1 and mCherry–Syn1 before the application of 7% 1,6-hexanediol. Dotted lines indicate the cell boundary. Right, the same cell as left, but 30 s after the application of 7% 1,6-hexanediol. Scale bar, 10 µm. **b**, Line plots of fluorescence intensity from the transparent white line in **a** indicated before (green solid) and after (green dotted) the application of 7% 1,6-hexanediol. **c**, Left, example micrograph of a dendrite expressing GFP–Itsn1 and mCherry–Syn1 before the application of 7% 1,6-hexanediol. Right, the same dendrite as left, but 30 s after the application of 7% 1,6-hexanediol. Scale bar, 5 µm. **d**, Line plots of fluorescence intensity from the transparent white line in **b** indicated before (green solid) and after (green dotted) the application of 7% 1,6-hexanediol. **e**, Additional 3-color 2D STED representative images of side-view synapses stained for Bassoon, Itsn1 and PSD-95 as in Fig. 2. **f**, Additional 3-color 2D STED representative images of side-view synapses stained for Bassoon, EndoA1 and PSD-95 as in Fig. 2. **g**, Additional 3-color 2D STED representative images of side-view synapses stained for Bassoon, Syn1 and PSD-95 as in Fig. 2. **h**, Additional 3-color 2D STED representative images of side-view synapses stained for Bassoon and EndoA1 as in Fig. 2. Endogenous Itsn1 was stained by a primary anti-Itsn1 antibody and a secondary antibody conjugated to Atto647N. Colocalization of signals (coloc) is displayed as white. **i**, Axonal (orange) and synaptic (red) regions used for fluorescence intensity measurements in Fig. 2h,i. **j**, Pipeline schematic for synaptic vesicle (SV) and clathrin-coated vesicle (CCV) isolation used for Fig. 2j. Source data

To test whether Itsn1 forms condensates in synapses, we first applied two aliphatic alcohols, 7% 1,6-hexanediol and 7% 1,4-butanediol, which disrupt phase-separated droplets^36^, to neurons expressing GFP–Itsn1 (FL) and mCherry–Syn1 and measured the coefficient of variation (CV) of fluorescent signals along the axons to quantify condensate dispersion (Fig. 2a–d)^43^. Within 30 s, Itsn1 and Syn1 puncta in axons (Fig. 2a,c), the soma and dendrites (Extended Data Fig. 3a–d) of these neurons were markedly diffused. In agreement, measured CVs for both axonal GFP–Itsn1 and axonal mCherry–Syn1 signals were reduced after 1,6-hexanediol treatment or 1,4-butanediol treatment (Fig. 2b,d), indicating that condensed structures are formed via the weak hydrophobic interactions of these proteins.

To better understand the function of Itsn1 condensates in neurons, we performed three-color 2D stimulated emission depletion (STED) microscopy to localize Itsn1 relative to interacting partners and subsynaptic domains (Fig. 2e and Extended Data Fig. 3e–h). Given the functional interaction of Itsn1 and EndoA1 (refs. ^41,44,45^) and their cocondensation (Extended Data Fig. 2c), we visualized the relative locations of Itsn1 and EndoA1 to the reserve pool marked by Syn1, the active zone marked by Bassoon, and standardized our synapse assignment by costaining for the postsynaptic marker PSD-95 and visualizing only side-view synapses^33^. We performed endogenous staining with antibodies in differential combinations. Three-color 2D STED imaging revealed that Itsn1 and EndoA1 form small overlapping puncta at presynapses and that these puncta are located within and at the inside edge of Bassoon signals (Fig. 2e and Extended Data Fig. 3e–h). To understand the specific location of these overlapping Itsn1–EndoA1 puncta to subsynaptic domains, the distances of Itsn1, EndoA1 and Syn1 puncta to the midline of Bassoon signal were quantified. These data showed increasing average distances, respectively (Fig. 2f). EndoA1 and Itsn1 signals had no bias to the center of active zone (Fig. 2f). Cumulative frequency analysis further revealed that Itsn1 and EndoA1 puncta are shifted away from Syn1, sitting between the Bassoon and synapsin signals (Fig. 2g). Furthermore, given an average width of ~100 nm for Bassoon STED signal, roughly 63% of Itsn1 puncta and 47% of EndoA1 puncta fall within Bassoon signal (which extends ~50 nm out from the midline at 0 nm; green rectangle, Fig. 2g). Thus, Itsn1 and EndoA1 puncta sit within and at the edge of Bassoon signal, or the active zone, and sit below Syn1 signal, or the reserve pool, implicating these proteins in functions at or near the active zone.

We then tested whether these condensates undergo dispersion and reclustering during neuronal activity in a way that mirrors synaptic vesicle dynamics by expressing either GFP–Itsn1 or EndoA1–mRFP in mouse hippocampal neurons and following its distribution after 300 action potentials (APs) given at 10 Hz (Fig. 2h,i and Extended Data Fig. 3i). As in previous reports, which suggest endophilin disperses from synapses during activity^43^, these two proteins undergo dynamic dispersion and then recondense in response to neuronal activity (Fig. 2h,i and Extended Data Fig. 3i). Finally, to test if these proteins bind synaptic vesicles, we purified synaptic vesicles from mouse brains^53^ and probed for Itsn1 and EndoA1 using antibodies (Fig. 2j and Extended Data Fig. 3j). We used purified clathrin-coated vesicles as a control because Itsn1 and EndoA1 are found on these vesicles^53^. We found that both Itsn1 and EndoA1 are present on purified synaptic vesicles (Fig. 2j). Together, these data suggest that Itsn1 and EndoA1 form activity-responsive condensates adjacent to the active zone.

### Itsn1 maintains a vesicle pool for transient docking

**Fig. 3:**
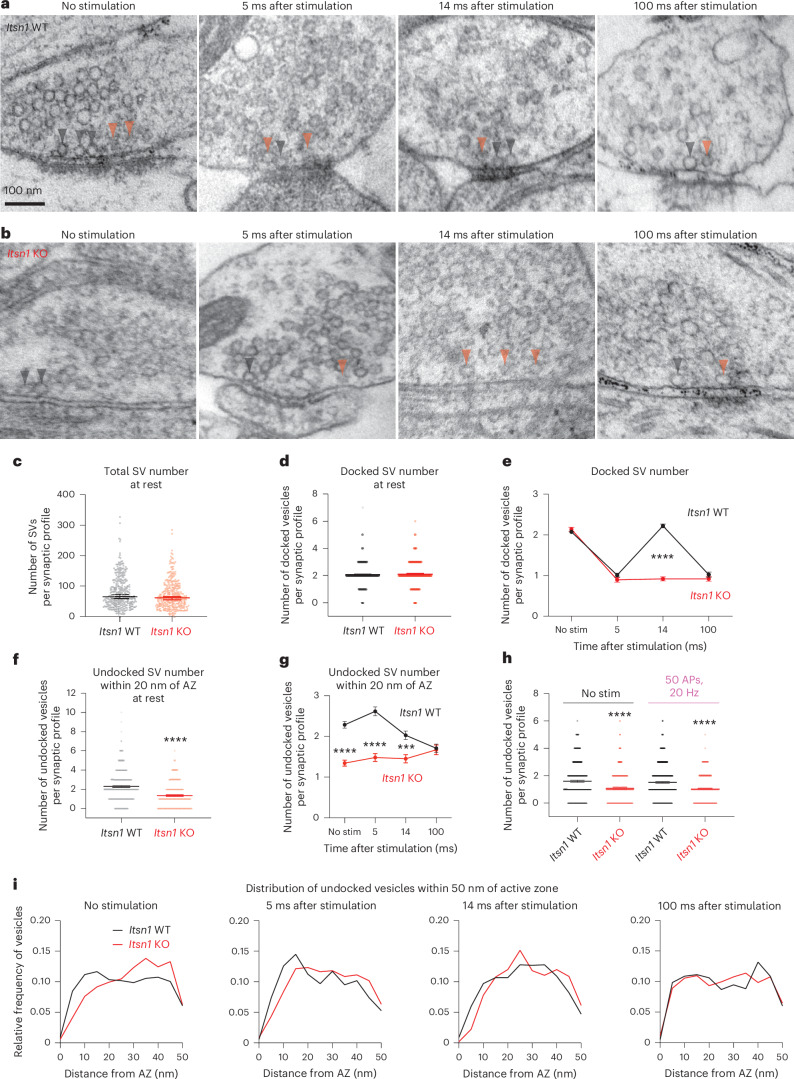
Itsn1 organizes undocked vesicles needed for transient docking. **a**, Electron micrographs showing the progression of docked and undocked vesicle abundance and localization at rest and at indicated time points following in *Itsn1*^*+/+*^ (WT). Black arrowhead, docked vesicle; orange arrowhead, undocked vesicle. Scale bar, 100 nm. **b**, Same in **a** for *Itsn1*^−*/*−^ (KO). **c**, Number of SVs in *Itsn1* WT (black) and *Itsn1* KO (red). Bars are the mean; error bars are s.e.m. **d**, The total number of docked vesicles at rest in *Itsn1* WT and *Itsn1* KO. Bars are the mean; error bars are s.e.m. **e**, Number of docked vesicles at rest and indicated time points after stimulation in *Itsn1* WT and *Itsn1* KO. Dots are the mean; error bars are s.e.m. Kruskal–Wallis test, with Dunn’s multiple comparisons test. ****P < 0.0001. Comparisons were made between *Itsn1* WT and *Itsn1* KO. **f**, Number of undocked vesicles within 20 nm of the AZ membrane at rest in *Itsn1* WT and *Itsn1* KO. Bars are the mean; error bars are s.e.m. Two-sided Mann–Whitney *U* test. ****P < 0.0001. **g**, Number of undocked vesicles within 20 nm of the AZ at rest and indicated time points after stimulation in *Itsn1* WT and *Itsn1* KO. Bars are the mean; error bars are s.e.m. Kruskal–Wallis test, with Dunn’s multiple comparisons test. ****P < 0.0001 and ***P = 0.0003. Comparisons were made between *Itsn1* WT and *Itsn1* KO. **h**, Number of undocked vesicles within 20 nm of the AZ in *Itsn1* WT and *Itsn1* KO synaptic profiles at rest or after 50 repetitive stimuli delivered at 20 Hz. Bars are the mean; error bars are s.e.m. Kruskal–Wallis test, with Dunn’s multiple comparisons test. ****P < 0.0001. Comparisons were made between *Itsn1* WT and *Itsn1* KO in no stimulation (left) or 50 AP, 20 Hz (right) conditions. **i**, Relative frequency distributions of undocked vesicles 2–50 nm from the AZ membrane. Vesicle counts were determined in *Itsn1* WT and *Itsn1* KO at rest and indicated time points after stimulation (left to right). Vesicle counts were separated into 2 nm bins and normalized by the total number of vesicles in this region. Analysis is done in synaptic profiles. See Supplementary Table 1 for additional information. AZ, active zone. Source data

**Extended Data Fig. 4:**
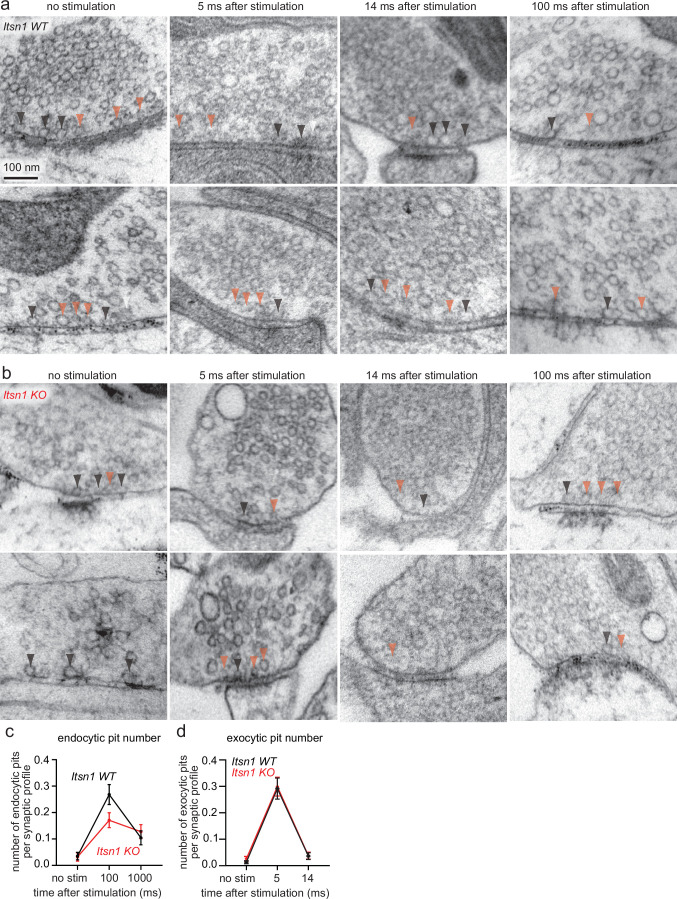
Itsn1 WT and KO membrane dynamics. **a**, Additional example electron micrographs from Fig. 3a. Scale bar, 100 nm. Black arrowhead: docked vesicle. Orange arrowhead: undocked vesicle. **b**, Additional example electron micrographs from Fig. 3b. Black arrowhead: docked vesicle. Orange arrowhead: undocked vesicle. **c**, Plots showing the number of endocytic pits in *Itsn1* WT (black) and KO (red) synaptic profiles at rest, 100 ms and 1000 ms after stimulation. Dots are the mean; error bars are s.e.m. **d**, Plots showing the number of exocytic pits in *Itsn1* WT (black) and KO (red) synaptic profiles at rest, 5 ms and 14 ms after stimulation. Dots are the mean; error bars are sem. Source data

Our data so far suggests that Itsn1 and EndoA1 form condensates near the active zone membrane and bind synaptic vesicles. To discern the synaptic function of these condensates, we conducted zap-and-freeze time-resolved electron microscopy experiments^7^. *Itsn1*^*+/+*^ (WT) and *Itsn1*^−*/*−^ (KO) mouse hippocampal neurons were frozen either unstimulated or stimulated with a 1-ms electrical pulse (see Methods for details), which induces a single AP, at various time points before freezing (Fig. 3 and Extended Data Fig. 4)^7^. Because Itsn1 was previously implicated in endocytosis in mouse hippocampal synapses^54^ and in the *Drosophila* neuromuscular junction^55^, we first quantified endocytic pit formation and resolution by stimulating *Itsn1* WT or *Itsn1* KO neurons and freezing 100 ms or 1 s after^5^. Consistent with recent work^48^, we found no substantial defect in ultrafast endocytosis in *Itsn1* KO synapses (Extended Data Fig. 4c).

To further probe the role of Itsn1 at synapses, we assessed vesicle dynamics at synapses. We defined vesicles touching the plasma membrane as docked, as in our previous studies^4,5,7^. In unstimulated conditions, *Itsn1* KO neurons had a normal number of docked and undocked vesicles (Fig. 3a–d and Extended Data Fig. 4a,b). To test whether these synapses functioned normally, we stimulated these neurons once and froze 5 ms after. Both *Itsn1* WT and *Itsn1* KO displayed exocytic pits at this time point (Extended Data Fig. 4d). Concomitantly, the number of docked vesicles was reduced at 5 ms (Fig. 3a,b,e and Extended Data Fig. 4a,b). Thus, exocytosis after a single stimulus is intact in *Itsn1* KO hippocampal synapses, consistent with previous studies in the calyx of Held synapses^46^.

We next observed docking dynamics after stimulation to test whether Itsn1 is involved in refilling release sites. Our previous studies suggest that vacated release sites can be replenished by ~14 ms after a single stimulus^7^, a process that may reflect synaptic plasticity and maintenance^20,21,24^. This docking process is reversible (hence, ‘transient docking’)—by 100 ms, the number of docked vesicles returns to the depleted state^4,5,7^. To test this functional paradigm in *Itsn1* KO synapses, we froze neurons 14 ms and 100 ms after a single stimulus. In *Itsn1* WT synapses, transient docking was normal—docked vesicles were recovered at 14 ms and depleted again by 100 ms (Fig. 3a,e and Extended Data Fig. 4a). By stark contrast, in *Itsn1* KO synapses, transient docking was completely abolished (Fig. 3b,e and Extended Data Fig. 4b). Interestingly, undocked vesicles in *Itsn1* KO synapses appeared scarce near the active zone, particularly in the first 20 nm (Fig. 3f). This ~35% reduction was present at baseline and persisted out to 14 ms after stimulation (Fig. 3f,g). Additionally, the distribution of undocked vesicles close to the active zone (within 50 nm) changed dynamically following an AP (Fig. 3i). In *Itsn1* WT, the number of undocked vesicles within 20 nm slightly increased at 5 ms after stimulation from baseline levels while slightly decreasing within 20–50 nm (Fig. 3g,i). This accumulation of undocked vesicles within 20 nm of the active zone then decreased at 14 ms as docked vesicle counts increased via transient docking (Fig. 3e,g,i). In resting *Itsn1* KO synapses, undocked vesicles were relatively more abundant between 20 and 50 nm of active zones (Fig. 3i), and redistribution of these vesicles into the 20 nm region was slow, requiring ~100 ms for *Itsn1* KO synapses to reach *Itsn1* WT levels (Fig. 3g,i). With repetitive stimulation (50 APs, 20 Hz) of *Itsn1* WT and *Itsn1* KO synapses, vesicles within 20 nm of the active zone are maintained, although at a lower level in *Itsn1* KO (Fig. 3h). Given the steady turnover of membrane-adjacent vesicles during continuous activity^56^, this maintenance indicates the 20 nm zone is refilled in both *Itsn1* WT and *Itsn1* KO, yet in the KO these vesicles never accumulate enough to reach WT levels (Fig. 3h). Taken together, these data suggest that a sufficient number of undocked vesicles must be held by Itsn1 within 20 nm of the active zone to be used for transient docking. Without Itsn1, these vesicles are not properly enriched, thus preventing their use. Hereafter, we will refer to these vesicles as replacement vesicles and this 20 nm region as the replacement zone.

### Itsn1–EndoA1 interaction is critical for vesicle replacement

**Extended Data Fig. 5:**
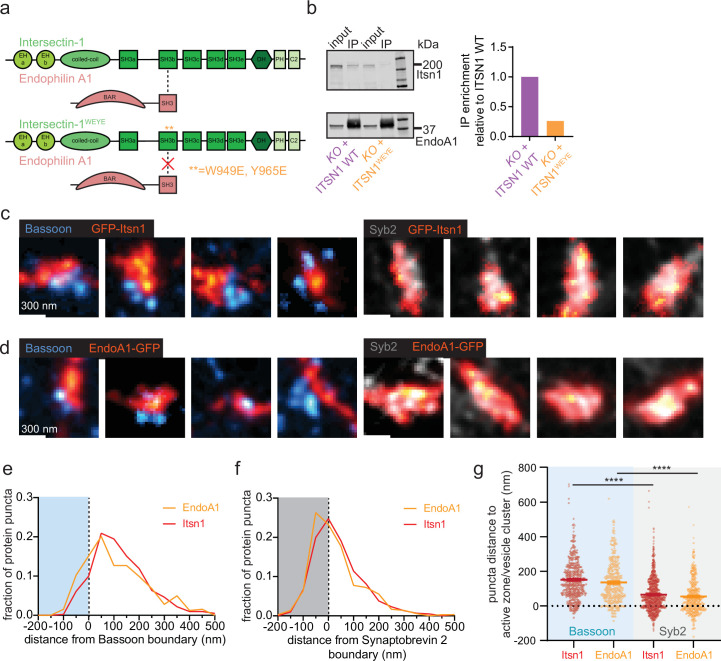
Point mutations block Itsn1 and EndoA1 binding in neurons. **a**, Top, schematic showing Itsn1 and EndoA1 domain structures with the Itsn1–EndoA1 binding site denoted between participating domains by a dotted line; bottom, binding prevented by W949E, Y965E mutations in Itsn1. **b**, Left, western blot showing the reduction of EndoA1 binding to Itsn1^WEYE^ by comparing EndoA1 pull down in either *Itsn1* KO^+^ ITSN1 WT or *Itsn1* KO^+^ ITSN1^WEYE^ lysates; right, quantification of IP enrichment relative to *Itsn1* KO^+^ ITSN1 WT. **c**, Example 2D STED images of different mouse hippocampal presynapses expressing GFP–Itsn1 showing its localization relative to the active zone, marked by Bassoon (cyan) or synaptic vesicles, marked by Syb2 (gray). GFP–Itsn1 was stained by an anti-GFP antibody and a secondary antibody conjugated to Atto647N. Endogenous Bassoon was used as a proxy for the active zone and was stained by an anti-Bassoon antibody and a secondary antibody conjugated to Alexa594. Endogenous Syb2 was stained by an anti-Syb2 antibody and a secondary antibody conjugated to Alexa594. Scale bar, 300 nm. **d**, Example 2D STED images of different mouse hippocampal presynapses expressing EndoA1–GFP showing its localization relative to the active zone, marked by Bassoon (cyan) or synaptic vesicles, marked by Syb2 (gray). EndoA1–GFP was stained by an anti-GFP antibody and a secondary antibody conjugated to Atto647N. Endogenous Bassoon was used as a proxy for the active zone and was stained by an anti-Bassoon antibody and a secondary antibody conjugated to Alexa594. Endogenous Syb2 was stained by an anti-Syb2 antibody and a secondary antibody conjugated to Alexa594. Scale bar, 300 nm. **e**, Cumulative plots showing the distribution of GFP–Itsn1 and GFP–EndoA1 puncta by 2D STED relative to the active zone boundary (dotted line), defined by Bassoon signal (blue fill). **f**, Cumulative plots showing the distribution of GFP–Itsn1 and GFP–EndoA1 puncta by 2D STED relative to synaptic vesicle localization boundary (dotted line), defined by Syb2 signal (gray). **g**, Plot showing distances between either GFP–Itsn1 or EndoA1–GFP puncta and either the active zone boundary or synaptic vesicle localization boundary. Bars are the mean; error bars are s.e.m. Kruskal–Wallis test, with Dunn’s multiple comparisons test. ****p < 0.0001. P values are shown for comparisons between both GFP–Itsn1 and EndoA1–GFP distances to Syb2 versus distances to Bassoon. See Supplementary Table 1 for additional information. Source data

Itsn1 and EndoA1 form condensates near the active zone and bind vesicles, and without Itsn1, the consequent reduction in replacement vesicles coincides with failure of transient docking. Recent work suggests that the interaction of Itsn1 and EndoA1 is critical for continued vesicle release in stimulated chromaffin cells^45^, highlighting the potential for neuronal vesicle replacement to have similar requirements. To test this possibility, we performed rescue experiments in *Itsn1* KO neurons with ITSN1 WT or the mutant (W949E/Y965E^41^; hereafter ITSN1^WEYE^) form of Itsn1, which prevents EndoA1 binding (Extended Data Fig. 5a,b)^41^.

To test whether expression of Itsn1 and EndoA1 recapitulates endogenous localization, mouse hippocampal neurons expressing Itsn1 or EndoA1 tagged with GFP were stained with an anti-GFP antibody together with either endogenous staining of the v-SNARE synaptobrevin-2 (Syb2) or Bassoon and imaged by two-color, 2D STED. Fluorescent puncta were quantified as a function of their distance from the boundary of either Bassoon or Syb2 signals^36^. These data suggest that a substantial fraction of overexpressed Itsn1 and EndoA1 proteins are contained within and next to the active zone, thus recapitulating endogenous distribution (Fig. 2e–g and Extended Data Fig. 5c–g). Additionally, costaining with Syb2 revealed that a large fraction of Itsn1 and EndoA1 is on or near synaptic vesicles (Extended Data Fig. 5c–g), further implicating these proteins in direct regulation of synaptic vesicle dynamics.

**Fig. 4:**
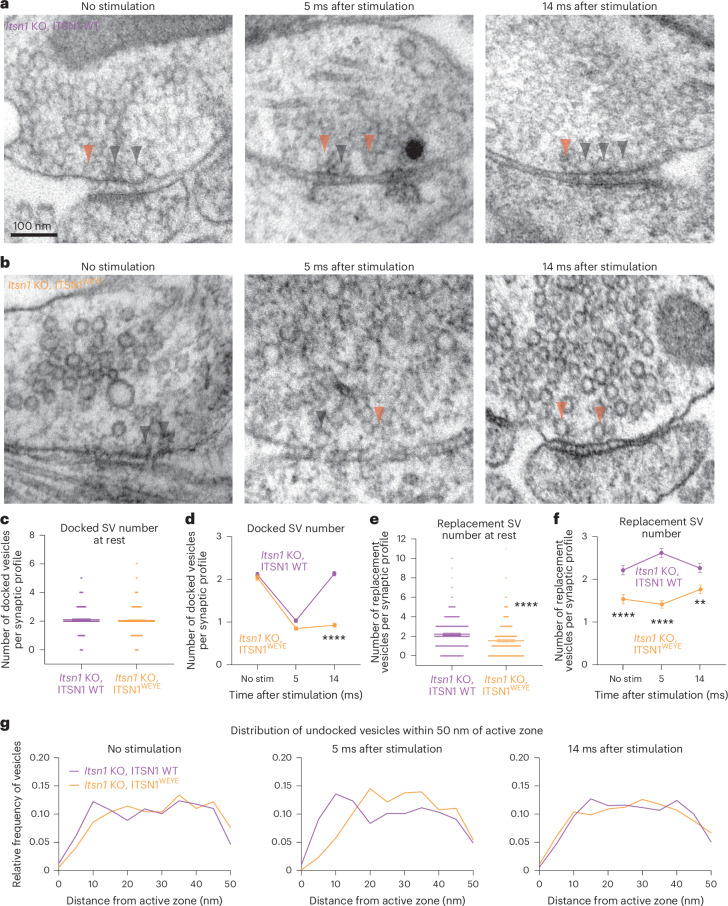
Interaction of Itsn1 and EndoA1 is required for transient docking. **a**,**b**, Electron micrographs showing the progression of docked vesicle and undocked vesicle abundance and localization in *Itsn1* KO neurons expressing ITSN1 WT (**a**) and ITSN1 W949E, Y965E mutant (ITSN1^WEYE^; **b**) at rest, 5 or 14 ms after stimulation. Black arrowhead, docked vesicle; orange arrowhead, undocked vesicle. Scale bar, 100 nm. **c**, Number of docked vesicles in *Itsn1* KO, ITSN1 WT and *Itsn1* KO, ITSN1^WEYE^ synaptic profiles at rest. Bars are the mean; error bars are s.e.m. **d**, Number of docked vesicles at rest, 5 and 14 ms after stimulation in *Itsn1* KO, ITSN1 WT and *Itsn1* KO, ITSN1^WEYE^. Bars are the mean; error bars are s.e.m. Kruskal–Wallis test, with Dunn’s multiple comparisons test. ****P < 0.0001. Comparisons were made between *Itsn1* KO, ITSN1 WT and *Itsn1* KO, ITSN1^WEYE^. **e**, Same as in **c**, but for replacement vesicles in the replacement zone (undocked vesicles within 20 nm of the active zone). Bars are the mean; error bars are s.e.m. Two-sided Mann–Whitney *U* test. ****P < 0.0001. **f**, Same as in **d**, but for replacement vesicles in the replacement zone. Bars are the mean; error bars are s.e.m. Kruskal–Wallis test, with Dunn’s multiple comparisons test. ****P < 0.0001 and **P = 0.0014. Comparisons were made between *Itsn1* KO, ITSN1 WT and *Itsn1* KO, ITSN1^WEYE^. **g**, Relative frequency distributions of undocked vesicles 2–50 nm from the active zone membrane in *Itsn1* KO, ITSN1 WT and *Itsn1* KO, ITSN1^WEYE^ at rest, 5 and 14 ms after stimulation (left to right). Vesicle counts were separated into 2 nm bins and normalized by the total number of vesicles in this region. Analysis is done in synaptic profiles. See Supplementary Table 1 for additional information. Source data

**Extended Data Fig. 6:**
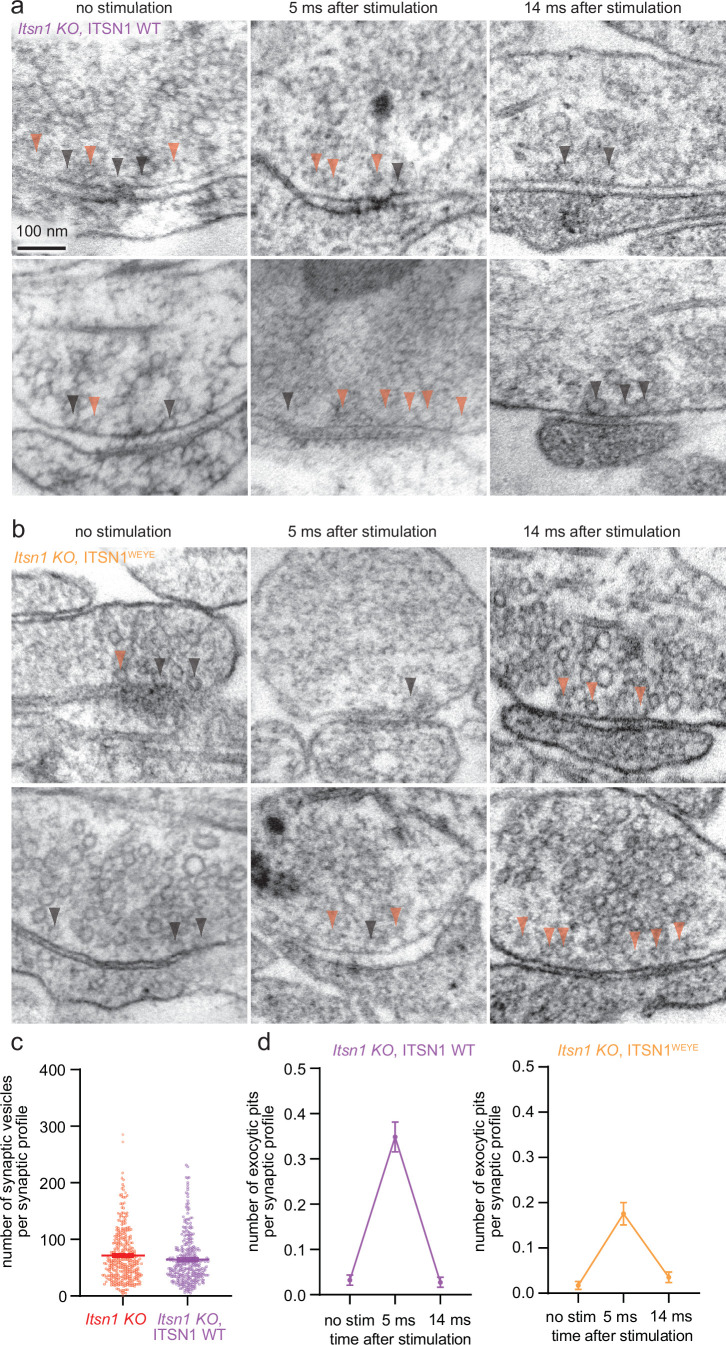
Itsn1 KO and rescue membrane dynamics. **a**, Additional example electron micrographs from Fig. 4a. Scale bar, 100 nm. Black arrowhead: docked vesicle. Orange arrowhead: undocked vesicle. **b**, Additional example electron micrographs from Fig. 4b. Black arrowhead: docked vesicle. Orange arrowhead: undocked vesicle. **c**, Number of synaptic vesicles per synaptic profile in *Itsn1* KO (red) and *Itsn1* KO, ITSN1 WT (purple) neurons. Bars are the mean; error bars are s.e.m. **d**, Plots showing the number of exocytic pits in *Itsn1* KO, ITSN1 WT (purple, left) and *Itsn1* KO, ITSN1^WEYE^ (orange, right) synaptic profiles at rest, 5 ms and 14 ms after stimulation. Dots are the mean; error bars are s.e.m. See Supplementary Table 1 for additional information. Source data

At the ultrastructural level, overall synapse morphology was normal, and the total number of vesicles was unperturbed relative to *Itsn1* KO when rescue constructs were expressed (Fig. 4a,b and Extended Data Fig. 6a–c). After a single AP, exocytosis occurred normally^7^ in rescue backgrounds (Extended Data Fig. 6d). When ITSN1 WT was expressed in *Itsn1* KO neurons, all phenotypes were rescued (Fig. 4a,c–g and Extended Data Fig. 6a). By contrast, the expression of ITSN1^WEYE^ failed to rescue the transient docking phenotype—docked vesicles were depleted at 5 ms normally but failed to return to baseline 14 ms after stimulation (Fig. 4b,c,d and Extended Data Fig. 6b). As in *Itsn1* KO, this failure coincided with a lack of replacement vesicles in the replacement zone (Fig. 4b,e,f and Extended Data Fig. 6b), and the distribution of replacement vesicles was shifted away from the active zone relative to ITSN1 WT rescue at all time points (Fig. 4g). Taken together, our results suggest that the mutation of Itsn1’s EndoA1 binding site perturbs the localization of replacement vesicles and their mobilization for transient docking.

### EndoA1 localizes Itsn1 to mobilize the replacement pool

**Fig. 5:**
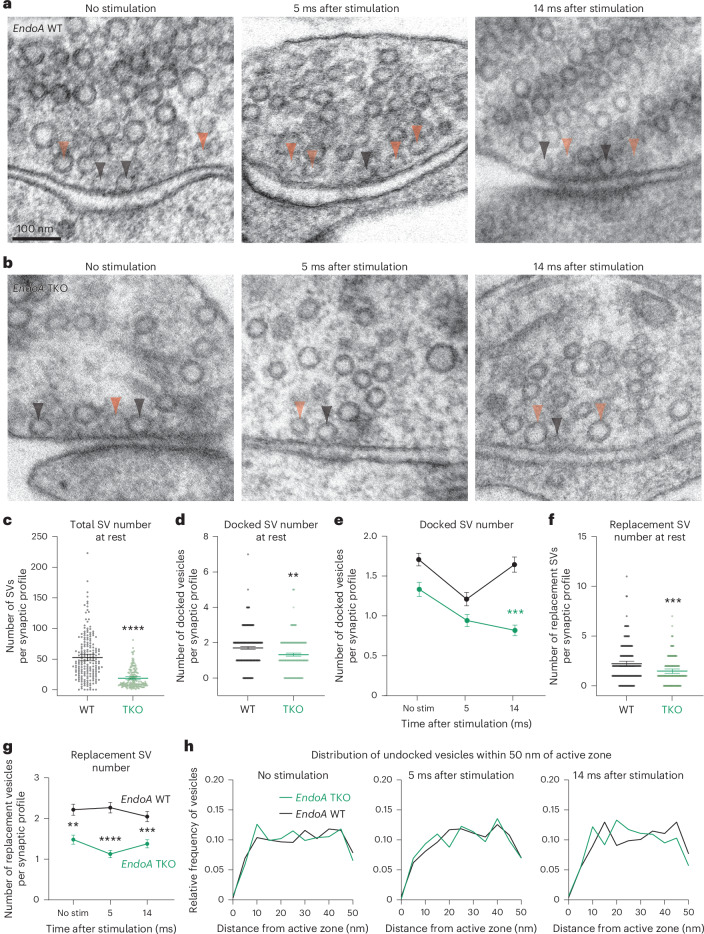
EndoA1 is required for transient docking of synaptic vesicles. **a**,**b**, Electron micrographs showing the progression of docked vesicle and undocked vesicle abundance and localization in *EndoA* WT (**a**) and *EndoA* TKO (**b**) synapses at rest, 5 ms and 14 ms after stimulation. Black arrowhead, docked vesicle; orange arrowhead, undocked vesicle. Scale bar, 100 nm. **c**,**d**, The total number of all vesicles in the terminal (**c**) and those docked in the active zone (**d**) at rest in *EndoA* WT (black) and TKO (green). Bars are the mean; error bars are s.e.m. Two-sided Mann–Whitney *U* test. ****P < 0.0001 (**c**) and **P = 0.0017 (**d**). **e**, Number of docked vesicles at rest, 5 and 14 ms after stimulation in *EndoA* WT and TKO. Bars are the mean; error bars are s.e.m. Kruskal–Wallis test, with Dunn’s multiple comparisons test. ***P = 0.0002. Comparison was made between *EndoA* TKO no stimulation and TKO 14 ms after stimulation. **f**, Number of replacement vesicles in the replacement zone at rest in *EndoA* WT and TKO. Bars are the mean; error bars are s.e.m. Two-sided Mann–Whitney test. ***P = 0.0001. **g**, Number of replacement vesicles in the replacement zone at rest and 5 or 14 ms after stimulation in *EndoA* WT and TKO synaptic profiles. Bars are the mean; error bars are s.e.m. Kruskal–Wallis test, with Dunn’s multiple comparisons test. **P = 0.002, ***P = 0.0008 and ****P < 0.0001. Comparisons were made between *EndoA* WT and TKO. **h**, Relative frequency distributions of undocked vesicles 2–50 nm from the active zone membrane in *EndoA* WT and TKO synaptic profiles at rest, 5 and 14 ms after stimulation (left to right). Vesicle counts were separated into 2 nm bins and normalized by the total number of vesicles in this region. Analysis is done in synaptic profiles. See Supplementary Table 1 for additional information. Source data

**Extended Data Fig. 7:**
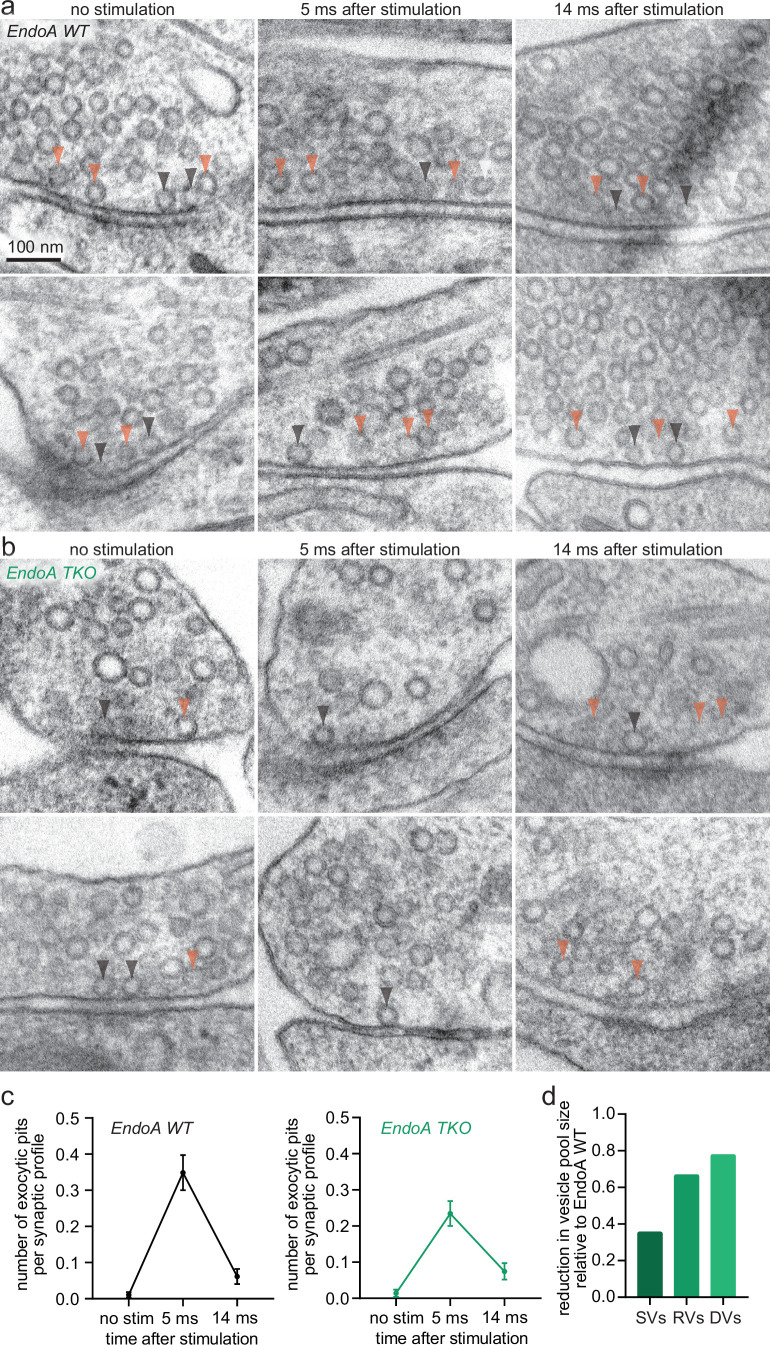
EndoA1 TKO membrane dynamics. **a**, Additional example electron micrographs from Fig. 5a. Scale bar, 100 nm. Black arrowhead: docked vesicle. Orange arrowhead: undocked vesicle. **b**, Additional example electron micrographs from Fig. 5b. Black arrowhead: docked vesicle. Orange arrowhead: undocked vesicle. **c**, Plots showing the number of exocytic pits in *EndoA1* WT (black, left) and *EndoA1* TKO (green, right) synaptic profiles at rest, 5 ms and 14 ms after stimulation. Dots are the mean; error bars are s.e.m. **d**, Bar graph showing the reduction in indicated vesicle pool sizes when calculating the ratio of vesicle counts between *EndoA* TKO and WT. SVs, synaptic vesicles; RVs, replacement vesicles; DVs, docked vesicles. See Supplementary Table 1 for additional information. Source data

Given its colocalization with Itsn1 in synapses and on vesicles and the importance of the Itsn1–EndoA1 interaction in replacement vesicle localization and transient docking, we next assessed the contribution of EndoA directly to this replacement pathway. We compared endophilin A (EndoA) triple KO (TKO; *Sh3gl2*^−/−^*, Sh3gl1*^−/−^ and *Sh3gl3*^−/−^) mouse hippocampal neurons to *EndoA* WT neurons by zap-and-freeze. As in our previous studies^57^, the number of synaptic vesicles was reduced in *EndoA* TKO neurons by ~70% due to the defect in synaptic vesicle recycling^57^ (Fig. 5a,b,c and Extended Data Fig. 7a,b). Despite this strong reduction in total vesicles, the number of docked vesicles and replacement vesicles at rest was only modestly decreased (~30%; Fig. 5a,b,d,f and Extended Data Fig. 7a,b,d), suggesting that remaining vesicles in *EndoA* TKO relatively accumulate near the active zone. The number of exocytic pits also decreased by ~30% when compared to *EndoA* WT, but the frequency of their appearance still fell within the normal range^7^ (Extended Data Fig. 7c).

*EndoA* WT synapses displayed normal transient docking (Fig. 5a,e and Extended Data Fig. 7a) and a subtle increase in replacement vesicles at 5 ms that returned to baseline at 14 ms (Fig. 5a,g and Extended Data Fig. 7a), mirroring transient docking. Strikingly, despite the relative accumulation of vesicles near the active zone (Extended Data Fig. 7d) in *EndoA* TKO synapses, transient docking completely failed (Fig. 5b,e and Extended Data Fig. 7b), suggesting that the absolute reduction in replacement vesicles (Fig. 5f) and lack of EndoA1 protein are sufficient to prevent transient docking. Furthermore, following stimulation of *EndoA* TKO synapses, replacement vesicles slightly decrease at 5 ms and return to baseline at 14 ms (Fig. 5b,g and Extended Data Fig. 7b), likely mirroring the failure of these vesicles to be mobilized for transient docking. Concomitantly, the relative distribution of undocked vesicles in *Endo* TKO neurons was similar to that in WT neurons in both unstimulated synapses and those frozen 5 ms or 14 ms after stimulation (Fig. 5h), suggesting that remaining undocked vesicles are properly localized, yet unavailable for replacement.

**Extended Data Fig. 8:**
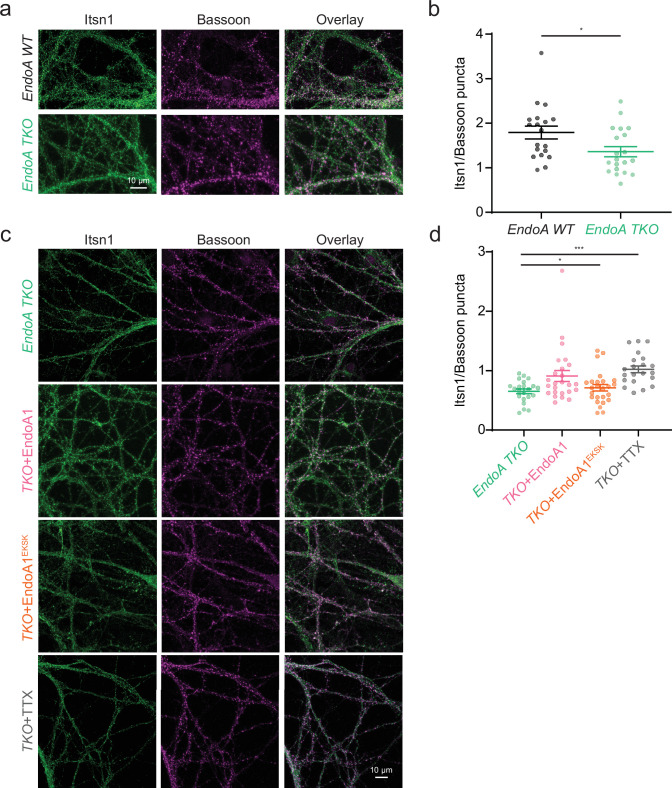
EndoA1 localizes Itsn1 near the active zone. **a**, Immunofluorescence of intersectin-1 and Bassoon in *EndoA* WT and *EndoA* TKO hippocampal neurons. Scale bar 10 µm. **b**, Quantification of Itsn1 intensity at Bassoon puncta in *EndoA* WT and *EndoA* TKO neurons. Bars are the mean; error bars are s.e.m. Two-sided Student’s t test. *p = 0.0239. **c**, Immunofluorescence of intersectin-1 and Bassoon in *EndoA* TKO hippocampal neurons compared to *EndoA* TKO expressing full-length endophilin A1 (TKO + EndoA1), intersectin-1 binding mutant EndoA1 (TKO + EndoA1^EKSK^), or *EndoA* TKO cells treated with TTX (TKO + TTX). TTX (1 µm, 14–16 h) was used to silence activity in TKO cultures. Scale bar 10 µm. **d**, Quantification of Itsn1 intensity at Bassoon puncta in the different conditions. Bars are the mean; error bars are s.e.m. One-way ANOVA with Dunnett’s multiple comparison test. *p = 0.0194, ***p = 0.0008. Source data

To better understand why remaining replacement vesicles were insufficient to induce transient docking, we visualized Itsn1 in *EndoA* TKO mouse hippocampal neurons and observed a decrease in Bassoon-overlapping Itsn1 puncta compared to WT neurons (Extended Data Fig. 8a,b). Expression of EndoA1, which rescues *EndoA* TKO recycling defects and thus restores vesicle abundance^57^, rescues the active zone-adjacent localization of Itsn1 (Extended Data Fig. 8c,d). However, expression of an otherwise normal EndoA1 that cannot bind Itsn1 (EndoA1 E329K, S336K^41^, here EndoA1^EKSK^) fails to rescue Itsn1 localization (Extended Data Fig. 8c,d), indicating that vesicle abundance in the terminal alone does not rescue Itsn1 positioning. Interestingly, applying tetrodotoxin (TTX) to *EndoA* TKO neurons rescues Itsn1 localization, likely due to the prolonged lack of vesicle use, suggesting that Itsn1 can eventually localize to the active zone-adjacent to the undocked vesicle pool, but in active synapses requires recruitment by EndoA1 binding for efficient localization (Extended Data Fig. 8c,d). Together, these data suggest that EndoA1 localizes Itsn1 to the replacement zone at a speed and to a level necessary to activate resident undocked vesicles for replacement.

### Replacement vesicles are necessary for short-term plasticity

**Fig. 6:**
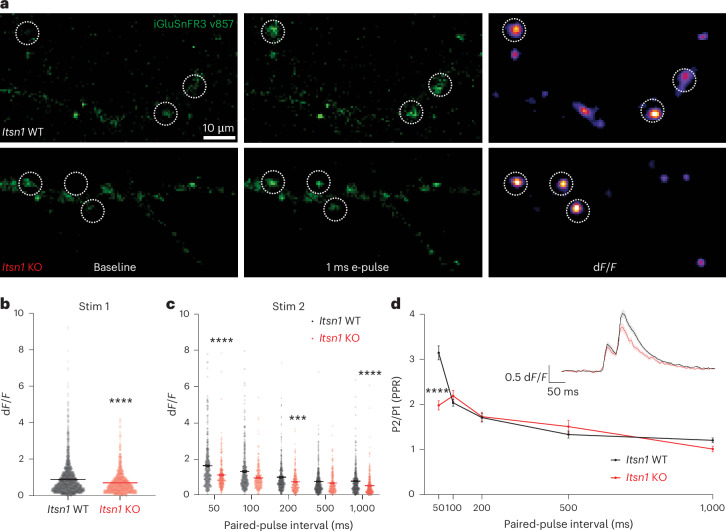
Itsn1 is required for glutamate release and synaptic facilitation. **a**, Representative images of *Itsn1* WT (top) or *Itsn1* KO (bottom) neurons expressing iGluSnFR3 v857. Left images show neuronal processes at baseline. Middle images show neurons directly after a 1-ms e-pulse was applied to cells. Right shows the same regions transformed to show peak d*F*/F values. Synaptic regions of interest (ROIs) are shown as a dotted circle. Scale bar, 10 µm. **b**, Peak iGluSnFR d*F*/*F* values resulting from glutamate release from a single stimulation in either *Itsn1* WT or *Itsn1* KO neurons. Bars are the mean; error bars are s.e.m. Two-sided Mann–Whitney test. ****P < 0.0001. **c**, Peak iGluSnFR d*F*/*F* values resulting from glutamate release from the second stimulation in a paired-pulse experiment. Paired-pulse intervals range from 50 ms to 1,000 ms (1 s). Bars are the mean; error bars are s.e.m. Kruskal–Wallis test, with Dunn’s multiple comparisons test. ***P = 0.0003 and ****P < 0.0001. Comparisons were made between *Itsn1* WT and KO cells at 50 ms, 200 ms and 1,000 ms, respectively. **d**, PPR plotted for paired-pulse experiments conducted at varying paired-pulse intervals, ranging from 50 ms to 1,000 ms (1 s). PPRs are quantified as the peak *dF*/F value from stimulation 2 (P2) divided by the peak d*F*/F value from stimulation 1 (P1). Dots are the mean; error bars are s.e.m. Kruskal–Wallis test, with Dunn’s multiple comparisons test. ****P < 0.0001. Inset, representative d*F*/*F* traces from either *Itsn1* WT or *Itsn1* KO cells taken from a paired-pulse experiment with a 50 ms time interval. Lines are an average of all release site responses; error bars are s.e.m. See Supplementary Table 1 for additional information. Source data

To assess the importance of replacement vesicles for synaptic physiology, we expressed a third-generation variant of the intensity-based glutamate-sensing fluorescent reporter (iGluSnFR), iGluSnFR3 v857, which is sensitive, bright and shows rapid on kinetics for glutamate binding at excitatory synapses^58^. We expressed this probe in *Itsn1* WT and KO mouse hippocampal neurons and applied electric field stimulation (e-pulse) for 1 ms to engender glutamate release (Fig. 6a). We then measured changes in fluorescence (d*F*/*F*) in response to stimulation in synaptic regions (Fig. 6a). Both *Itsn1* WT and KO neurons respond to stimulation, although *Itsn1* KO neurons show a slightly reduced glutamate release (~20%; Fig. 6b).

**Extended Data Fig. 9:**
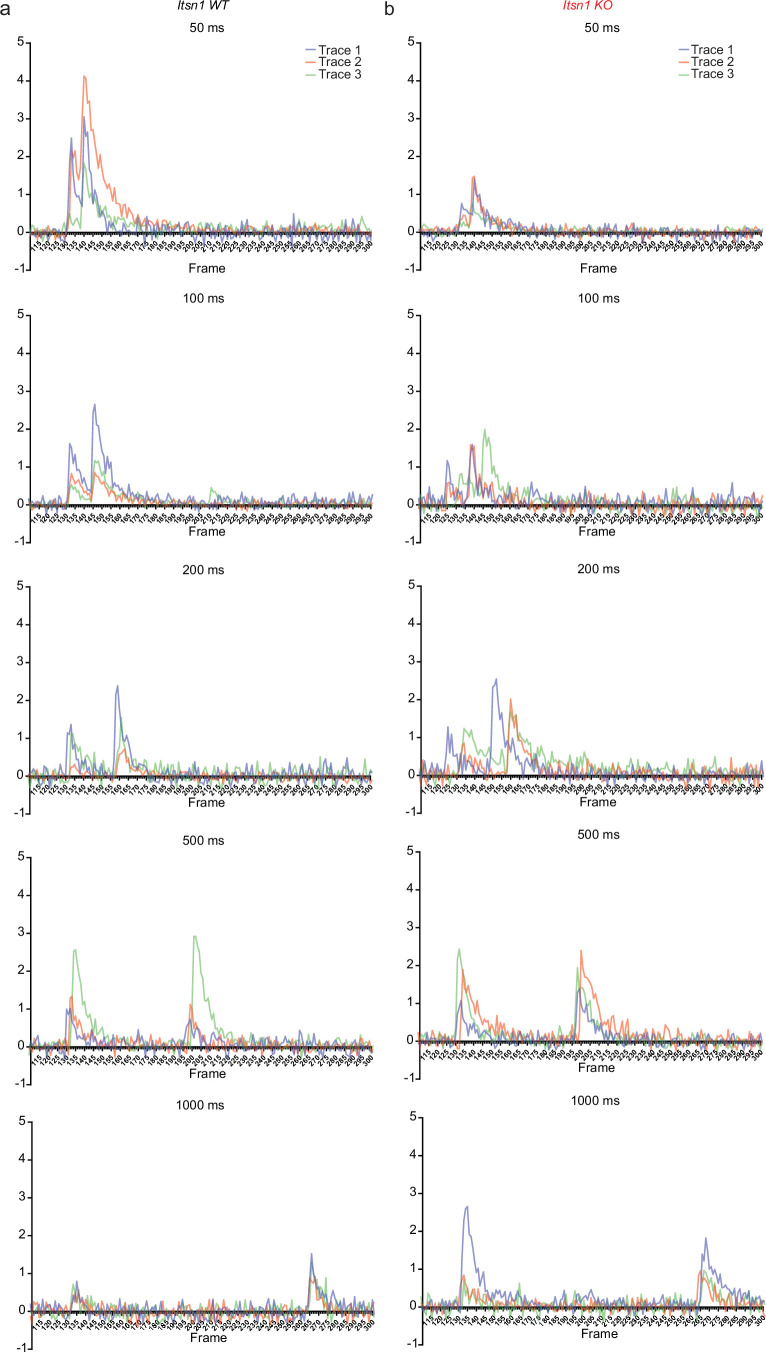
Itsn1 WT and KO iGluSnFR traces. **a**, Representative dF/F traces taken from iGluSnFR3 v857-expressing *Itsn1* WT neuron release sites. Traces from three release sites are shown (red, green and blue) across paired-pulse experiments with noted time intervals. **b**, Representative dF/F traces taken from iGluSnFR3 v857-expressing *Itsn1* KO neuron release sites. Traces from three release sites are shown (red, green and blue) across paired-pulse experiments with noted time intervals. Source data

Our previous studies show the following two major physiological deficits in the absence of transient docking: no facilitation of synaptic transmission and faster synaptic depression^7,21^. Thus, we measured synaptic facilitation by applying two e-pulses as close as 50 ms and as far as 1 s apart and quantified the ratio (paired-pulse ratio (PPR)) between the two responses. In *Itsn1* WT neurons, glutamate release increased in response to the second e-pulse when stimulations were paired closely in time (up to ~200 ms; Fig. 6c,d). In *Itsn1* KO neurons, the second response was reduced by nearly 40% from *Itsn1* WT levels when the second pulse was spaced 50 ms from the first (Fig. 6c and Extended Data Fig. 9a,b), resulting in a much lower PPR (Fig. 6d and Extended Data Fig. 9a,b). Furthermore, in these *Itsn1* KO neurons, glutamate release remained reduced at all subsequent paired-pulse intervals (Fig. 6c). Taken together, *Itsn1* KO neurons showed reduced synaptic facilitation and thus much faster depression.

### Replacement vesicles underlie brain slice neurotransmission

**Fig. 7:**
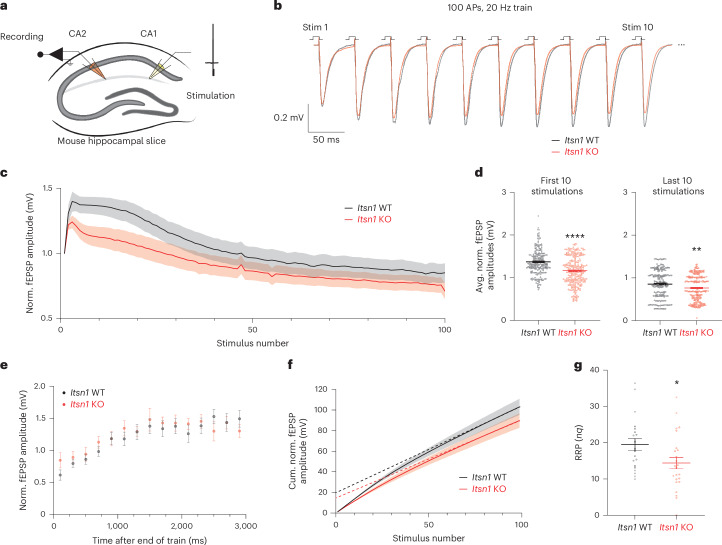
*Itsn1* KO displays enhanced short-term depression and RRP size reduction in adult brain slices. **a**, Schematic representation of mouse hippocampal slice fEPSP recording procedure. **b**, Average traces of the fEPSP response to the first ten stimulations from a 100 AP, 20 Hz train applied to either *Itsn1* WT (black) and KO (red) slices. **c**, Normalized fEPSP response amplitudes through the course of the train stimulation in *Itsn1* WT and KO slices. Transparent fill around traces is the s.e.m. **d**, The average of normalized fEPSP amplitudes from the first ten stimulations (left) and the last ten stimulations (right) in *Itsn1* WT and KO slices. Bars are the mean; error bars are s.e.m. Two-sided Student’s *t*-test. ****P < 0.0001 and **P = 0.0023. **e**, Normalized fEPSP amplitude following 100 AP, 20 Hz trains in *Itsn1* WT and KO slices, showing synaptic recovery. Recovery stimulations were initially applied 100 ms after the end of the train. Then, 200 ms were added sequentially to the time interval between the end of the train and the recovery stimulation out to 2,900 ms. Dots are the mean, and error bars are s.e.m. **f**, Cumulative normalized fEPSP amplitudes during 100 AP, 20 Hz trains in *Itsn1* WT and KO slices. Dotted lines are linear regression analyses. Transparent fill around traces is the s.e.m. **g**, RRP sizes (*nq*) in synapses from *Itsn1* WT and KO slices, approximated by linear back-extrapolation from traces in **f**. Two-sided Student’s *t*-test. *P = 0.0351. Bars are the mean; error bars are s.e.m. See Supplementary Table 1 for additional information. Norm., normalized; avg., average; cum., cumulative. Source data

**Extended Data Fig. 10:**
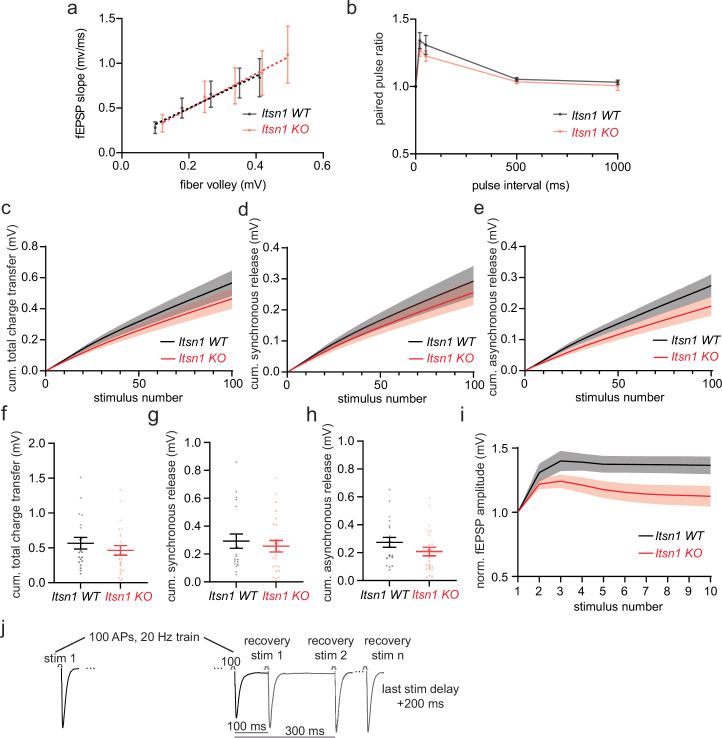
Itsn1 WT and KO slice recordings reveal neurotransmitter release differences. **a**, Release probability in *Itsn1* WT and *Itsn1* KO slices measured by comparing the fEPSP slope to fiber volley amplitudes in response to stimulations of increasing mV size (20 mV, 40 mV, 60 mV, 80 mV and 100 mV*)*. fEPSPs, field excitatory postsynaptic potentials. Dotted lines are linear regression analyses. Error bars are s.e.m. **b**, Plot showing paired-pulse ratio in *Itsn1* WT and *Itsn1* KO slices. A second pulse was separated by 20 ms, 50 ms, 500 ms and 1 s after the first pulse. The paired-pulse ratio was determined by dividing the peak of the second pulse by the initial pulse peak. Dots are the mean; error bars are s.e.m. **c**, Cumulative (cum.) total charge transfer (mV) measured from field excitatory postsynaptic potentials (fEPSPs) in response to stimulation; 100 AP, 20 Hz train. Transparent fill around traces is the s.e.m. **d**,**e**, Cum. total charge transfer from a, separated by the first 10 ms from each pulse (**d**) and the next 40 ms (**e**). The first 10 ms is defined as synchronous, while the next 40 ms is asynchronous. Transparent fill around traces is the s.e.m. **f**, Last cum. total charge transfer values measured from fEPSPs in response to a 100 AP, 20 Hz train. Bars are the mean, and error bars are s.e.m. **g**, Last cum. synchronous (first 10 ms of release per stimulation) charge transfer values measured from fEPSPs in response to a 100 AP, 20 Hz train. Bars are the mean, and error bars are s.e.m. **h**, Last cum. asynchronous (last 40 ms of release per stimulation) charge transfer values measured from fEPSPs in response to a 100 AP, 20 Hz train. Bars are the mean, and error bars are s.e.m. **i**, Normalized fEPSP amplitude from the first 10 pulses during the train. Transparent fill around traces is the s.e.m. **j**, Experimental paradigm schematic of synaptic recovery experiments in Fig. 7e. Following 100 AP, 20 Hz trains, single recovery stimulations were applied first at 100 ms after the train, then 300 ms and then with delays increasing by 200 ms every next recovery stimulation n. Source data

Finally, we measured field excitatory postsynaptic potentials (fEPSPs) in mouse hippocampal slices taken from *Itsn1* WT and KO by stimulating the CA1 region and recording from CA1/CA2 synapses in the CA2 region^59^ (Fig. 7a) to assess the importance of replacement vesicles in a more intact system. Unlike our findings by iGluSnFR, which show a slight reduction in instantaneous glutamate release in *Itsn1* KO neurons (Fig. 6b), there was no difference in initial release probability between WT and *Itsn1* KO slices (Extended Data Fig. 10a). To measure synaptic facilitation, we applied paired-pulse stimulations to *Itsn1* WT and KO slices. Similar to our release probability measurement, *Itsn1* KO showed only a slight reduction in paired-pulse responses compared to WT (Extended Data Fig. 10b). This is likely because the activity-dependent docking factor Syt7 is abundant in these synapses^21,60^, recruited synapses may not fully rely on Itsn1 or some level of adaptation to *Itsn1* KO has occurred. To assess whether *Itsn1* KO slices may eventually show faster synaptic depression than WT, we applied a high-frequency stimulation (100 APs, 20 Hz) and measured the continued synaptic response (Fig. 7b,c). In line with our iGluSnFR experiments (Fig. 6c,d), facilitation early in the train (first ten stimulations) was greatly reduced in *Itsn1* KO slices, as reflected by reduced average fEPSP amplitudes (Fig. 7b–d and Extended Data Fig. 10i). This reduction led to a faster depression of synaptic signaling (Fig. 7c and Extended Data Fig. 10i), suggesting that Itsn1-controlled replacement vesicles are still needed for brain slice synapses to keep up with repetitive signaling demands. Later in the train (last ten stimulations), the responses were more similar but still significantly different between WT and *Itsn1* KO, with *Itsn1* KO responses being smaller (Fig. 7c,d). Given that this phase represents the balance between exocytosis and replenishment, Itsn1 is likely to also have a significant role in steady-state replenishment. In agreement, there was an appreciable reduction of overall release, synchronous and asynchronous release during the train measured by charge transfer in *Itsn1* KO (Extended Data Fig. 10c–h). Despite these defects, synaptic recovery after the train was indistinguishable between WT and KO (Fig. 7e and Extended Data Fig. 10j), suggesting *Itsn1* KO synapses can steadily return to baseline release competency.

**Fig. 8:**
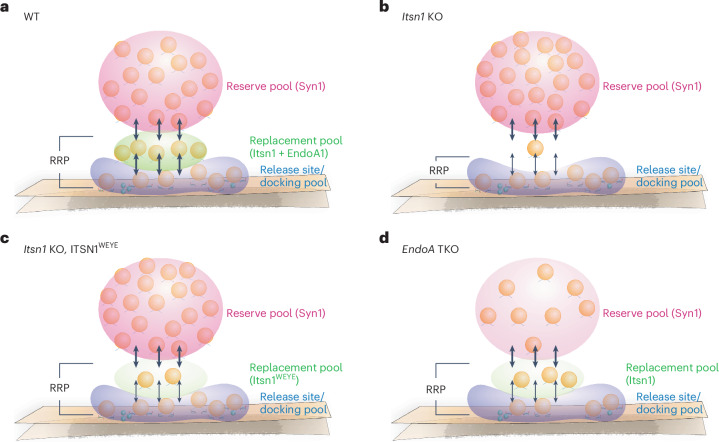
A new model for release site replenishment. **a**, Schematic representation showing a new model of activity-dependent replenishment of release sites during sustained neurotransmission and short-term plasticity. The RRP consists of the docked vesicle pool and the replacement vesicle pool. The replacement vesicle pool is maintained by Itsn1 and EndoA1, while the reserve pool is maintained by Syn1. Following stimulation, the vacated release sites are rapidly replenished by replacement vesicles to enhance synaptic signaling. **b**, In the absence of Itsn1 condensates, the replacement pool is depleted, and replenishment fails due to lack of replacement vesicle substrate. **c**, With only Itsn1^WEYE^ present, the replacement pool is also diminished and dysfunctional. **d**, In *EndoA* TKO, the replacement pool becomes smaller and is bereft of Itsn1. As a result, vesicles are not mobilized for replenishment at the release site.

Finally, we calculated the RRP size by linear back-extrapolation of cumulative normalized fEPSP responses during train stimulation^61–63^. The RRP was reduced by 30% in *Itsn1* KO slices when compared to WT slices (Fig. 7f,g). This reduction was consistent with the ~35% reduction of replacement vesicles (Fig. 3h) and the ~40% reduction in the second iGluSnFR response after applying paired pulses separated by 50 ms (Fig. 6c). Taken together, these data suggest that replacement vesicles make a substantial contribution to the RRP, are mobilized after the initial round of release for replacement that underlies sustained neurotransmission and plasticity and fail to do so in the absence of Itsn1, EndoA1 or their interaction (Fig. 8).

## Discussion

In the work presented here, we find that Itsn1, EndoA1 and their binding are required to cluster a pool of replacement vesicles within the replacement zone via condensation and vesicle binding. These vesicles are used to replenish release sites via transient docking following single stimuli or a train of stimuli to enhance synaptic signaling (Fig. 8a). Without Itsn1 condensates in *Itsn1* KO, these replacement vesicles are mislocalized (Fig. 8b). In the ITSN1^WEYE^ rescue background, Itsn1 and EndoA1 binding is lost, thereby likely mislocalizing Itsn1 and leading to an inability to properly accumulate replacement vesicles (Fig. 8c). Finally, in *EndoA* TKO, Itsn1 is mislocalized, leading to a loss of replacement-competent vesicles (Fig. 8d). The abovementioned defects result in the failure of transient docking and thus sustained synaptic transmission and facilitation.

We describe a zone driven by condensation of Itsn1 and EndoA1, positioned between active zone proteins and the Syn1-driven reserve pool of synaptic vesicles. Our data here suggest that the RRP of hippocampal excitatory synapses is further divided into the docked vesicle pool and the replacement pool (Fig. 8). Consistent with this idea, morphological studies in hippocampal mossy fiber synapses suggest that if all vesicles within 60 nm of the active zone were to fuse, the amount of membrane deposited into the presynaptic membrane would mirror the amount of membrane added during RRP-depleting stimulation, which was determined by capacitance measurements^25,64^. These molecular layers, and thereby functional pools, likely retain their identity in resting synapses due to the presence of interfacial tension imposed by phase separations. The changes of electrical potential, such as during elevation of calcium during synaptic activity, can potentially break these interfaces^65^ either directly by dissolving the phase separation or indirectly by post-translational modifications (as in CaMKII phosphorylation of synapsin^37^), enabling more vesicles to be available commensurate with the activity level of synapses. In fact, recent work has implicated the scaffolding protein Piccolo in freeing vesicles from the reserve pool by a phase separation mechanism^34^, which may enable capturing of those vesicles by the nearby Itsn1–EndoA1 system and ultimately enable a transition to the active zone upon dispersion. Thus, these molecular condensates can also regulate the number of vesicles available for release at a given synapse.

Furthermore, interactions with specific physical domains or structures within the presynapse may bias the localization of proteins that would otherwise bind each other and potentially cocondensate. For example, Itsn1 has long been shown to interact with various membrane-associated proteins in the active zone, like SNAP-25 (refs. ^48,66^). This may work to anchor Itsn1, and thus Syn1, along with their associated vesicle pools, at presynaptic terminals in a specific order from the active zone membrane. Finally, actin filaments may act as scaffolds on which condensates and their organelles bind, a model supported by the presence of actin in the reserve pool, active zone and active zone-adjacent region where many condensed proteins reside^67–70^. When these actin filaments are disrupted, release site replenishment fails^22,23^, and in recent work, this failure is compounded by simultaneous addition of 1,6-hexanediol^71^, indicating that actin filaments may work in conjunction with presynaptic phases like those formed by Itsn1 to regulate vesicle dynamics. Interestingly, Itsn1 regulates the actin cytoskeleton via its Dbl homology domain^72^, which has been implicated in release site replenishment^46,73^. Thus, the interaction between Itsn1 condensates and actin filaments in synaptic vesicle dynamics is an exciting avenue of investigation.

## Methods

### Animal use

All the animal work was performed according to the National Institutes of Health guidelines for animal research with approval from the Animal Care and Use Committees at the Johns Hopkins University School of Medicine. For *Itsn1* KO experiments, 129SV/J^Itsn1−^ mice were maintained as heterozygotes, and neurons were cultured from homozygous null P0 pups, with homozygous WT littermates used as controls. Slices or whole brains were taken from homozygous null or homozygous WT mice 6–8 weeks of age. For experiments not concerning *Itsn1* KO, neurons were cultured from E18 embryos from C57BL/6J mice (WT; Jackson Laboratory).

For the *EndoA* KO experiments, all procedures complied with national animal welfare regulations and were approved by the Institutional Animal Care Board of the University Medical Center Göttingen and the Lower Saxony State Office for Consumer Protection and Food Safety. Constitutive KO mice for EndoA1, EndoA2 and EndoA3, as originally described^47^, were used in the following two separate breeding schemes: (1) EndoA1^−/−^EndoA2^−/−^ (hereafter 1,2 DKO; lethal by P19–20) and littermate A1^+/−^A2^+/−^ mice were obtained from breeding A1^+/−^A2^+/−^ mice, and (2) perinatally lethal EndoA1^−/−^EndoA2^−/−^EndoA3^−/−^ (hereafter TKO) and littermate A1^−/−^A2^+/+^A3^−/−^ mice were obtained from breeding A1^−/−^A2^+/−^A3^−/−^ mice. WT controls were obtained from breeding EndoA1^+/−^EndoA2^+/−^EndoA3^+/−^ (genetic background ~80% C57BL/6J/~20% SV129) mice until EndoA1^+/+^EndoA2^+/+^EndoA3^+/+^was achieved or are obtained from the C57BL/6J line. Genotyping was performed as in ref. ^47^. Both male and female mice were used for all experiments.

### Primary neuron culture

Primary hippocampal neurons were isolated from either E18 embryos or P0 pups of both sexes. The brains were taken from animals, and the hippocampi were dissected under a binocular microscope. Dissected hippocampi were collected in ice-cold dissecting media (1× HBSS (Gibco, 14175095), 1 mM sodium pyruvate (Gibco, 11360070), 10 mM HEPES (Gibco, 15630080) pH 7.2–7.5, 30 mM glucose (Sigma, G6152-100G), 1% penicillin–streptomycin (Gibco, 15140122)) and later digested with 0.5 mg ml^−1^ papain (Worthington, LS003119) and 0.01% DNaseI (Sigma, DN25) for 25 min at 37 °C. Cells were then further dissociated by trituration using fire-polished Pasteur pipettes.

For high-pressure freezing experiments, neurons were plated onto 6-mm sapphire disks (Technotrade, 616-100) coated with 1 mg ml^−1^ poly-d-lysine (PDL; Sigma, P6407) and 0.6 mg ml^−1^ collagen (Thermo Fisher Scientific, 720081) with a preseeded astrocyte feeder layer on it. For this, cortices were collected from E18/P0 animals, and astrocytes were isolated with a treatment of 0.05% trypsin–EDTA (Sigma, T1426) for 20 min at 37 °C, followed by trituration. Astrocytes were seeded in T75 flasks (Sarstedt, 100437) coated with PDL containing DMEM (Gibco, 10569010) supplemented with 10% FBS (Thermo Fisher Scientific, 26140079) and 0.2% penicillin–streptomycin. After 2 weeks, astrocytes were plated on sapphire disks (50k per well). After 1 week in culture, astrocytes were incubated with 81 µM 5-fluoro-2′-deoxyuridine (Sigma, F0503) and 204 µM uridine (Sigma, U3003) for at least 2 h to stop mitosis. Before the addition of hippocampal neurons, the medium was changed to neurobasal-A (Gibco, 10888022) supplemented with 2 mM GlutaMax (Gibco, 35050061), 2% B27 (Gibco, 17504044) and 0.2% penicillin–streptomycin.

For high-pressure freezing experiments of *EndoA* TKO neurons and their controls, the following protocol was used. Astrocyte feeder cells were prepared as detailed in ref. ^74^. Hippocampi from transgenic animals were dissected and incubated for 25 min in 1× HBSS with 0.5% papain at 37 °C. After washing, neurons were triturated with fire-polished Pasteur pipettes, counted with a hemacytometer and plated on astrocyte microislands^75^ (the plating medium, neurobasal medium (Thermo Fisher Scientific, 21103049), supplemented with B27, 17.3 mm HEPES, 1% GlutaMax, 1% penicillin–streptomycin (Invitrogen), 25 μM β-mercaptoethanol (2-βME; Thermo Fisher Scientific, 21985023) and 100 nM insulin)^76^. Medium was exchanged after about 12 h with neuronal medium. Neurons were cultured for 12–14 days before being used for experiments (only islands containing single neurons were examined). Dissociated cultures of primary cortical neurons have been prepared as previously described^47^.

For fluorescence imaging, dissociated hippocampal neurons were seeded onto 18-mm or 25-mm coverslips (Carolina Biologicals, 633013 and 633017) coated with 1 mg ml^−1^ poly-l-lysine (Sigma, P2636) at a density of 25–40 × 10^3^ cells cm^−2^ in neurobasal medium supplemented with 2 mM GlutaMax, 2% B27, 5% FBS and 1% penicillin–streptomycin (NM5) at 37 °C in 5% CO_2_. The next day, the media was changed to neurobasal media with 2 mM GlutaMax and 2% B27 (NM0), and neurons were maintained in this medium until use. Half of the media was refreshed every week or as needed.

For iGluSnFR imaging, neurons were cultured as mentioned above for fluorescence imaging; however, on days in vitro (DIV) 4, 8, 11 and 14, half media swaps were conducted with glia-conditioned media (GCM). GCM was generated by adding 30 ml of NM0 to confluent astrocyte T75 flasks. After 2 days, GCM was collected, filtered and collected for use.

For biochemical experiments, dissociated hippocampal neurons were seeded on poly-l-lysine (1 mg ml^−1^) coated plates or dishes with neurobasal medium supplemented with 2 mM GlutaMax, 2% B27, 5% FBS and 1% penicillin–streptomycin, at a density of 1 × 10^5^ cells cm^−2^. The next day, the medium was changed to neurobasal medium containing 2 mM GlutaMax and 2% B27 (NM0), and neurons were maintained in this medium. Half of the media was refreshed every week or as needed. For *Itsn1* KO and *Itsn1* WT littermate cultures, tail clips were obtained from live P0 pups and genotyped as previously described^54^. Brain tissues were collected from correct genotypes, and hippocampal neurons were prepared as described above.

### Plasmids

For rescue experiments, rescue protein-coding sequences were recombined by in-fusion seamless cloning into a lentiviral expression vector containing 3× NLS–EGFP under the control of a human synapsin promoter. Downstream of the 3× NLS–EGFP is a P2A sequence, which lies directly upstream of the destination for rescue protein-coding sequences. Plasmids containing either FL Itsn1 isoform or the same isoform containing W949E and Y965E (WEYE) mutations were generated.

For localization experiments, a plasmid containing the FL Itsn1 isoform N-terminally tagged to GFP (GFP–Itsn1) was purchased from Addgene (47395). For EndoA1, an EndoA1 protein-coding sequence, which was a gift from the M.A. Cousin Lab, was dropped into a pEGFP-N1 CMV mammalian expression vector (Clontech) by in-fusion cloning, resulting in EndoA1 C-terminally tagged with EGFP (EndoA1–GFP).

For aliphatic alcohol experiments in neurons, GFP–Itsn1 was used with co-expression markers mCherry–Syn1.

For heterologous cell system experiments, we used the plasmids described above along with mammalian expression vectors containing mCerulean–Endo A1, BFP–Syn1, GFP–Itsn1 FL, HaloTag–Itsn1 FL, mCherry–Itsn1 AB, mCherry–Itsn1 A–E, untagged Syph and Syph–emiRFP670, cloned and/or maintained in the Milovanovic Lab.

For iGluSnFR experiments, a plasmid containing the iGluSnFR3.v857.GPI variant was purchased from Addgene (178331).

### Lentivirus preparation

Lentivirus containing either ITSN1 WT, ITSN1^WEYE^ or EndoA1^EKSK^ rescue constructs was prepared as described previously^36,59^. Briefly, either rescue construct along with two helper DNA constructs (pHR‐CMV8.2 deltaR (Addgene 8454) and pCMV‐VSVG (Addgene 8455)) at a 4:3:2 molar ratio was transfected into HEK293T cells (ATCC, CRL-3216) using polyethylene amine. Culture supernatant containing the virus was collected 3 days after transfection and 20-fold concentrated using Amicon Ultra 15 10K (Millipore, 901024) centrifugal filters. Aliquots were flash-frozen in liquid nitrogen and stored in −80 °C until use.

### Lentivirus infection

Neuron cultures prepared for lentiviral infection were grown until DIV 4–7. Titered lentivirus was added to the wells at a concentration that resulted in nearly 100% infection efficiency. For Itsn1 rescue experiments, successful infection was assayed by the expression of NLS–EGFP, which is contained within rescue constructs. Additionally, western blot analysis was used to assess the expression of the rescue construct protein of interest.

### Transient transfection

For transient expression of proteins, neurons were transfected at DIV 9–16 by Lipofectamine 2000 (Invitrogen, 11668030) according to the manufacturer’s instructions. Before transfection, half of the media from each well was removed and mixed with fresh NM0 (see above), which was then warmed to 37 °C and equilibrated with CO_2_ in an incubator (recovery media). The rest of the media was aspirated and replaced with fresh NM0 for transfection. Plasmids were diluted in NeuroBasal Plus (Gibco, A3582901) media so that 1–2 µg of DNA would be added to each well. Before the addition, DNA was mixed with a solution containing Lipofectamine 2000 such that there was a 1:1 to 1:4 ratio of µg DNA to µl Lipofectamine. This mixture was added to each well and incubated for 4 h. Afterward, the transfection media were removed and replaced with recovery media that had been previously prepared. After 16 h to 1 week, neurons were either used for pharmacological treatment or fixed for immunofluorescence.

For transient transfection of iGluSnFR, neurons were transfected at DIV 8–10 by Lipofectamine 2000 according to the manufacturer’s manual. Before transfection, half of the media from each well was removed and mixed with fresh GCM (see above), which was then warmed to 37 °C and equilibrated with CO_2_ in an incubator (recovery media). The rest of the media was aspirated and replaced with fresh NM0 for transfection. Plasmids were diluted in NeuroBasal Plus media so that 2 µg of DNA would be added to each well. Before the addition, DNA was mixed with a solution containing Lipofectamine 2000 such that there was a 1:3 ratio of µg DNA to µl Lipofectamine. This mixture was added to each well and incubated for 3 h. Afterward, the transfection media were removed and replaced with recovery media that had been previously prepared. After 6–8 days, neurons were used for iGluSnFR experiments.

Transient expression of proteins in HEK293T/HEK293 (ATCC, CRL-1573) cells occurred at most 1 day before experiments. Cells were grown on 35-mm glass-bottom dishes until 80% confluency. A solution of Lipofectamine 2000 and DNA (3 µl:1 µg) in 200 µl Opti-MEM (Gibco, 31985062) was prepared according to the manufacturer’s instructions. In total, 0.5 µg of DNA was used. The solution was mixed and allowed to sit for 20 min before being added to the cells. Cells were incubated for 12–24 h after addition at 37 °C and 5% CO_2_ before imaging.

### Pharmacology

For 1,6-hexanediol (Sigma, 240117) and 1,4-butanediol (Sigma, 493732) experiments in neurons, transfected neurons were treated immediately before imaging. Neurons plated on 25-mm coverslips were mounted onto a metal ring sample holder containing three-fourths of the final volume of cell culture medium. Upon initiation of the imaging experiment, the remaining one-fourth of the cell culture medium was added from a stock solution that contains either 28% 1,6-hexanediol or 1,4-butanediol to make a final concentration of 7%.

For HEK293T cells, cells were plated onto 35-mm glass-bottom Petri dishes (Cellvis, D35-20-1.5H). Dishes contained three-fourths of the final volume of the cell culture medium. Upon initiation of the imaging experiment, the remaining one-fourth of the cell culture medium was added from a stock solution that contains 16% 1,6-hexanediol to make a final concentration of 4% 1,6-hexanediol.

For HEK293 cell experiments, prewarmed 1,6-hexanediol was diluted to a final concentration of 3% in DMEM (culture media) and loaded onto cells plated on 35-mm glass-bottom Petri dishes.

For *EndoA* TKO immunofluorescence experiments, TTX (Tocris, 1078) was added at a concentration of 1 µM for 14–16 h.

### Immunofluorescence

For immunofluorescence, experiments were performed with DIV 14–16 hippocampal neurons. Culture media was removed from the wells and fixed with 37 °C 1× PBS (Thermo Fisher Scientific, 14190144) containing 4% paraformaldehyde (Electron Microscopy Sciences, 15714) and 4% sucrose (Sigma, S0389) for 20 min at room temperature. After fixation, cells were washed three times with 1× PBS. Next, cells were permeabilized with 0.2% Triton X-100 (Sigma, T9284) diluted in 1× PBS for 8 min. After three washes with 1× PBS, cells were blocked with 1% BSA (Sigma, A3294) in 1× PBS for 1 h. Then, coverslips were transferred to a humidified chamber and placed face down on a drop of primary antibody solution. Primary antibodies were diluted 1:500 to 1:250 in a 1% BSA 1× PBS, and cells were incubated at 4 °C overnight. GFP–Itsn1 and EndoA1–GFP were stained with an anti-GFP rabbit polyclonal antibody (MBL International, 598). Endogenous Bassoon protein was stained by an anti-Bassoon mouse monoclonal antibody (Synaptic Systems, 141011, or Enzo Life Sciences, SAP7F407). Endogenous synapsin protein was stained by an anti-Syn1/anti-Syn2 guinea pig polyclonal antibody (Synaptic Systems, 106004). Endogenous Syb2 protein was stained by an anti-Syb2 mouse monoclonal antibody (Synaptic Systems, 104211). Endogenous EndoA1 was stained by an anti-endophilin 1 guinea pig polyclonal antibody (Synaptic Systems, 159004). Endogenous Itsn1 was stained by an anti-Itsn1 rabbit polyclonal antibody (gift from V. Haucke). Next, coverslips were washed thrice with 1× PBS. Secondary antibodies were diluted in 1× PBS containing 1% BSA. For super-resolution two-color 2D STED imaging, an anti-rabbit or mouse Atto647N (Rockland Immunochemicals, 611-156-122S or 610-156-121S) secondary antibody was used at 1:120 dilution, and an anti-mouse, rabbit or guinea pig Alexa594 (Invitrogen, A11005, A11012 and A11076) secondary antibody was used at 1:1,000. For three-color 2D STED, the same secondary antibodies used in two-color 2D STED experiments were used, as well as an anti-mouse secondary antibody conjugated to STAR 460L (Aberrior, ST460L-1001) at 1:150 dilution and a FluoTag-2x nanobody conjugated to Atto643 raised against PSD-95 (NanoTag Biotechnologies, N3702-At643-L). Secondary antibody incubation was performed in a humidified chamber as described previously for 1 h at room temperature. Following three 1× PBS washes, cells were rinsed with MilliQ water and mounted on a glass slide containing a drop of ProLong Diamond Antifade Mounting Media (Thermo Fisher Scientific, P36970). Mounting media was allowed to solidify for 24 h at room temperature in the dark before proceeding to STED imaging.

For *EndoA* TKO neurons, cells were washed in prewarmed PBS and fixed in 4% paraformaldehyde containing 1% sucrose in PBS at 4 °C overnight or at room temperature for 30 min. The free aldehyde was quenched with 50 mM NH_4_Cl (Sigma, A6141) for 20 min at room temperature. Furthermore, the cells were permeabilized (0.1% Triton X-100) and blocked for 1 h in blocking solution (3% BSA, 0.1% cold fish gelatin (Sigma, G7041) and 1% goat serum (Sigma, G9023) in PBS). Bassoon and Itsn1 were labeled in the neurons with primary antibodies mentioned above in blocking buffer at 4 °C overnight, washed thrice for 10 min and corresponding species secondary antibodies (Alexa488—Invitrogen, A11001; Alexa647—Invitrogen, A21244) for 1 h at room temperature in dark conditions. The samples were washed thrice for 10 min in PBS to remove nonbound signal and then embedded on glass slides using Mowiol 4-88 mounting medium (Carl Roth, 0713.2).

### STED

Two-color 2D STED images were obtained using a home-built two-color STED microscope^36^. A femtosecond laser beam with a repetition rate of 80 MHz from a Ti:Sapphire laser head (Mai Tai HP; Spectra-Physics) is split into the following two parts: one part is used to produce the excitation beam, which is coupled into a photonic crystal fiber (Newport) for wide-spectrum light generation and is further filtered by a frequency-tunable acoustic optical tunable filter (AA Opto-Electronic) for multicolor excitation. The other part of the laser pulse is temporally stretched to ~300 ps (with two 15-cm-long glass rods and a 100-m-long polarization-maintaining single-mode fiber; OZ Optics), collimated, expanded and wave-front modulated with a vortex phase plate (VPP-1, RPC photonics) for hollow STED spot generation to de-excite the fluorophores at the periphery of the excitation focus, thus improving the lateral resolution. The STED beam is set at 765 nm with a power of 120 mW at the back focal plane of the objective lens (numerical aperture (NA) = 1.4 HCX PL APO ×100; Leica), and the excitation wavelengths are set as 594 nm and 650 nm for imaging Alexa594- and Atto647N-labeled targets, respectively. The fluorescent photons are detected by two avalanche photodiodes (SPCM-AQR-14-FC; PerkinElmer). Before imaging, lasers were aligned by TetraSpeck multicolor nanobeads. The images are obtained by scanning a piezo-controlled stage (Thorlabs, Max311D) controlled by the Imspector v16.3 data acquisition program.

Three-color 2D STED imaging was conducted on the Aberrior Facility Line microscope with standard settings, alignment and imaging parameters.

### Data analysis of 2D STED images

A custom MATLAB code package was used to analyze overexpressed GFP-tagged Itsn1 and EndoA1 protein distribution relative to the active zone marked by Bassoon and synaptic vesicle pools marked by Syb2 in 2D STED images^36^. First, STED images were blurred with a Gaussian filter with a radius of 1.2 pixels to reduce the Poisson noise and then deconvoluted twice using the built-in deconvblind function—the first point spread function (PSF) input is measured from nonspecific antibody signal in the STED images, and the second PSF input is chosen as the returned PSF from the first run of blind deconvolution^36^. Each time, ten iterations are performed. Presynaptic boutons in each deconvoluted image were selected within 30 × 30 pixel (0.81 mm^2^) regions of interest (ROIs) based on the varicosity shape and bassoon signal. The active zone or synaptic vesicle pool boundary was identified as the contour that represents half of the intensity of each local intensity peak in the Bassoon and Syb2 channels, respectively, and the Itsn1 or EndoA1 protein foci are picked as local maxima. The distances between the protein foci centers (*n*) and the active zone or synaptic vesicle pool boundary are automatically calculated correspondingly. Itsn1 and EndoA1 protein foci/puncta were continuous with the edge of ROIs, and the Bassoon or Syb2 signals outside of the transfected neurons were excluded from the analysis. As previously done, roughly 100 boutons were quantified from two different cultures (*n*) for each condition^36^. The MATLAB scripts are available by request.

For three-color 2D STED images, a custom MATLAB code package (https://github.com/imotolab-neuroem/STED_image_analysis_package_public_v1.4/tree/main) was used to analyze side-view synapses. As for two-color 2D STED images, images were deconvoluted as above after exporting from Aberrior Imspector (Abberior Instruments Development Team, Imspector Image Acquisition & Analysis Software v16.3, http://www.imspector.de). Presynaptic ROIs were 40 × 40 pixels (800 nm × 800 nm). Bassoon ‘bars’ were picked by Bassoon signal (with opposing PSD-95 signal), and a midline was drawn along the long axis. Puncta of proteins (*n*) of interest were identified as mentioned above, and distances to the Bassoon midline or the midpoint of that midline were quantified. For each condition, roughly 80 boutons were quantified from three to four different cultures (*n*) as above.

### Live-cell Airyscan imaging and data analysis

For Airyscan imaging, samples were imaged in Zeiss LSM880 (Carl Zeiss) in Airyscan mode. For 1,6-hexanediol or 1,4-butanediol experiments in neurons, fluorescence was acquired using a ×63 objective lens (NA = 0.55) at 488 × 488 pixel resolution and a pinhole size above the lower limit for Airyscan imaging, as computed by ZEN software (blue version, Zeiss). Neurons transfected with GFP–Itsn1 and mCherry–Syn1 on DIV 9 were imaged at DIV 14–16 before the addition of aliphatic alcohols. The field of view depended on the size of the neuron imaged. Full Z-stacks were acquired. Afterward, aliphatic alcohols were added to the imaging chamber, and immediately after, neurons were imaged by full Z-stack imaging to assess the dispersion of molecular condensates. For analysis, images were background-corrected using a rolling ball radius of 50 pixels. Then, a 1.0 sigma Gaussian blur was applied. After, lines with a pixel width of 10 were drawn parallel to the orientation of axons across condensates in Fiji. Intensity values for both GFP–Itsn1 and mCherry–Syn1 were measured before and after aliphatic alcohol addition. Mean intensity and intensity variance were measured along axons and used to calculate the CV by dividing the variance by the mean. CV values were normalized by the average CV value of axons measured in each condition, and the number of measurements was chosen as reported previously^43^.

For 1,6-hexanediol experiments in HEK293T cells, fluorescence was acquired using a ×63 objective lens (NA = 0.55) at 1,024 × 1,024 pixel resolution with the following settings: pixel dwell 0.24 µs and pinhole size above the lower limit for Airyscan imaging, as computed by ZEN software. Cells were transfected with GFP–Itsn1 and imaged following successful expression. Cells were recorded at 2 Hz for 1 min. At 30 s, 1,6-hexanediol was added to measure the dispersion of molecular condensates. Condensate–condensate fusion, if recorded before 1,6-hexanediol addition, was isolated and assessed separately.

Internal FRAP experiments were conducted in cells similarly prepared for 1,6-hexanediol experiments. Fluorescence was acquired using a ×63 objective lens (NA = 0.55) at 1,024 × 1,024 pixel resolution with the following settings: pixel Dwell 0.24 µs and pinhole size above the lower limit for Airyscan imaging, as computed by ZEN software. Cells were recorded with an exposure of 600 ms for 100 frames, for a total imaging time of ~1 min. After frame 3, Itsn1 condensates were bleached with a 488 laser initially at a diameter of 800 nm and then at 1600 nm. The recovery of fluorescence was measured throughout the course of the experiment in an appropriate number of condensates and cells, as previously reported^36^. Intensity values were transformed into fractional recovery over time to the maximum intensity value after bleaching from the initial intensity value following bleaching at frame 4. To measure the Tau of fluorescence recovery, fractional recovery values were fit by a nonlinear one-phase association function as previously reported^36^.

### Fixed-cell Airyscan imaging and data analysis

The Zeiss LSM800, fitted with an inverted stand (Axio Observer 7), was used for image acquisition from fixed samples. Plan Apo-Chromat differential interference contrast (DIC) ×63/1,4 oil was used, and the sample was illuminated by transmitted light (Halogen) with manual DIC, fluorescence (HBO (H, Hg; B, high luminance; O, unforced cooling) 100) and diode lasers (405, 488, 561 and 647 nm). Cascadable nondescanned detectors with a photomultiplier tube were used for the detection of three to four fluorescence channels simultaneously across the complete wavelength range. Microscope was operated by ZEN software, and postacquisition image analysis and processing were done using ZEN (black version, Zeiss) and ImageJ software.

### Live-cell confocal imaging and data analysis

Live-cell confocal imaging and the 1,6-hexanediol assay in HEK293 cells were performed and quantified as described previously^77^. In short, HEK293 cells were plated onto 35-mm glass-bottom Petri dishes and transfected with plasmids as indicated in the text using Lipofectamine 2000. Prewarmed 1,6-hexanediol was diluted to a final concentration of 3% in DMEM (culture media) and loaded into the cells. Images were acquired using either an Andor iXon DU-888 X-9798 or a PCO.edge (SN:18500826) camera, mounted on a Nikon Eclipse Ti spinning-disk confocal microscope (CSU-X, Andor Revolution SD system), equipped with an OkoLab live-cell incubator (37 °C, 5% CO₂) and a PL APO ×60/1.4 NA oil immersion objective. Excitation wavelengths were as follows: 405 nm for BFP, 488 nm for GFP, 561 nm for mCherry and 640 nm for emiRFP. All images were analyzed with ImageJ (NIH).

### Live-cell imaging by spinning-disk confocal microscopy and data analysis

The dispersion of GFP-labeled Itsn1 and EndoA1–mRFP proteins was imaged using the custom-built spinning-disk confocal system and quantified as described previously^45^. Neurons with low fluorescence (protein expression) and coverslips with lower transfection efficiency were preferred. Clustering and dispersion were monitored upon field stimulation of neurons at 37 °C with 300 AP at 10 Hz in Tyrode buffer (119 mM NaCl, 5 mM KCl, 25 mM HEPES buffer, 2 mM CaCl_2_, 2 mM MgCl_2_ and 6 g l^−1^ glucose (pH 7.4)) containing 50 μM 2-amino-5-phosphonovaleric acid (Sigma, A8054) and 10 μm 6-cyano-7-nitroquinoxaline-2,3-dione (Sigma, C239) to block recurrent activity. Intensity changes from the ROI (marked around the centroid of fluorescence intensity in synaptic boutons and near boutons along the axon) in time-series images were analyzed using ImageJ software.

### High-pressure freezing

In total, 75K hippocampal neurons cultured on sapphire disks were frozen using a high-pressure freezer (EM ICE; Leica Microsystems). For functional assessment of *Itsn1* KO, *Itsn1* KO cells, along with WT littermates, were prepared. For rescue experiments, lentivirus was added to the KO wells to express Itsn1 rescue constructs. For functional assessment of *EndoA1* KO, *EndoA* TKO cells were plated with WT littermates. For some experiments, 2 mg ml^−1^ ferritin (Sigma, F7879) was used as a fluid-phase marker and added to the cells for 5 min before freezing. Cells were frozen in a physiological saline solution (140 mM NaCl, 2.4 mM KCl, 10 mM HEPES, 10 mM glucose; pH adjusted to 7.3 with NaOH, 300 mOsm) containing NBQX (3 µM; Tocris, 1044) and bicuculline (30 µM; Tocris, 0109), which were added to block recurrent synaptic activity. CaCl_2_ and MgCl_2_ concentrations were adjusted as needed for experiments. Experiments conducted with *Itsn1* WT/KO neurons with or without rescue constructs were conducted with 4 mM CaCl_2_ and 1 mM MgCl_2_, while experiments conducted with *EndoA* WT/TKO neurons were conducted with 1.2 mM CaCl_2_ and 3.8 mM MgCl_2_. Zap-and-freeze experiments were performed as described earlier^7^. After freezing, samples were transferred under liquid nitrogen to an automated freeze-substitution system at −90 °C (EM AFS2; Leica Microsystems). Using precooled tweezers, samples were quickly transferred to anhydrous acetone at −90 °C. After disassembling the freezing apparatus, sapphire disks containing cells were quickly moved to cryo-baskets containing freeze-substitution solutions and left inside EM AFS2. Freeze substitution was performed in solutions containing 1% glutaraldehyde (Electron Microscopy Sciences, 16530) and 0.1% tannic acid (Sigma, 403040) in anhydrous acetone (solution A) and then 2% osmium tetroxide (Electron Microscopy Services, 19132) in anhydrous acetone (solution B), which had been stored under liquid nitrogen and then moved to the AFS2 immediately before use. The freeze-substitution program was as follows: −90 °C for 36 h in solution A, paused to swap to solution B after 6× washes for 30 min in −90 °C acetone, 5 °C h^−1^ to −20 °C, 12 h at −20 °C and 10 °C h^−1^ to 4 °C. Afterward, samples were removed from the freeze-substitution chamber and warmed at room temperature by 4× washes for 20 min with acetone before infiltration and embedding. For this latter protocol, all the steps were performed in universal sample containers (Leica Microsystems, 1670715 and 16707154) and kept covered with Aclar film to prevent any evaporation.

### Sample embedding and sectioning for electron microscopy

Following freeze substitution and washing, a 100% epon araldite (epon 6.2 g, araldite 4.4 g, DDSA 12.2 g and BDMA 0.8 ml; Ted Pella, 18012) solution was prepared and diluted with acetone to get 30%, 70% and 90% solutions. Samples were infiltrated for at least 2 h at room temperature sequentially in 30% and 70% Epon Araldite. Samples were then transferred to caps of polyethylene BEEM capsules (Electron Microscopy Sciences, 102096-558) with 90% epon araldite and incubated overnight at 4 °C. The next day, samples were transferred to new caps with fresh 100% Epon Araldite, changed every 2 h for three times, after which samples were cured at 60 °C for 48 h.

After the resin was cured, 40 nm sections were cut using an ultramicrotome (EM UC7; Leica Microsystems) and collected on single-slot copper grids (Ted Pella, 1GC12H) coated with 0.7% pioloform. The sections were then stained with 2.5 % uranyl acetate (Ted Pella, 19481) in a 70% methanol and 30% water solution.

### Electron microscopy imaging and data analysis

Samples were imaged on a Hitachi 7600 TEM equipped with an AMT XR50 camera run on AMT Capture v6 (pixel size = 560 pm) at 80 kV on the 100,000× setting. Samples were blinded before imaging. Synapses were identified by a vesicle-filled presynaptic bouton and a postsynaptic density. Postsynaptic densities are often subtle in our samples, but synaptic clefts were also identifiable by (1) their characteristic width, (2) the opposed membranes following each other closely and (3) vesicles near the presynaptic active zone. The 120–130 micrographs per sample of anything that appeared to be a synapse were taken without close examination. All images were from different synapses.

EM image analysis was performed as previously described^4,5,7,21,36,57,78,79^. All images from a single experiment were randomized for analysis as a single pool. Only after this randomization were any images excluded from analysis, either because they appeared not to contain a bona fide synapse or the morphology was too poor for reliable annotation. The plasma membrane, the active zone, exocytic and endocytic pits, clathrin-coated pits, docked synaptic vesicles and all synaptic vesicles in the bouton were annotated in ImageJ using SynapsEM plugins (https://github.com/shigekiwatanabe/SynapsEM (copy archived at) swh:1:rev:11a6227cd5951bf5e077cb9b3220553b506eadbe)^79^. To minimize bias and error and to maintain consistency, all image segmentation, still in the form of randomized files, was thoroughly checked and edited by a second member of the lab. Features were then quantified using the SynapsEM^79^ family of MATLAB (MathWorks) scripts (https://github.com/shigekiwatanabe/SynapsEM). Example electron micrographs shown were adjusted in brightness and contrast to different degrees (depending on the varying brightness and contrast of the raw images), rotated and cropped in ImageJ before being imported into Adobe Illustrator.

### Biochemical methods

#### Isolation of synaptic vesicles and clathrin-coated vesicles

Synaptic vesicles and clathrin-coated vesicles were isolated as reported previously^53^. Equal protein concentrations of synaptic and clathrin-coated vesicles were used for immunoblotting experiments.

#### Western blot analysis of vesicle isolates

Standard SDS–PAGE blot was used to analyze total protein levels. An electrophoresis system (Bio-Rad) was used to perform the separation using custom-prepared gels (4–15%, pH 8.8; Bio-Rad, 4561086), depending on the size of the protein to be analyzed by immunoblotting. After electrophoresis, the proteins were transferred onto a nitrocellulose membrane (Bio-Rad, 1620115) using the transfer system (Bio-Rad). The membranes were further blocked in 5% milk prepared in 1× Tris-buffered saline and 0.1% Tween 20 (TBS-T, blocking buffer) at room temperature for 1 h and subsequently incubated with primary (mouse monoclonal anti-clathrin heavy chain (HC; Abcam, Ab2731), mouse monoclonal anti-synaptophysin-1 (Synaptic Systems, 101011), mouse monoclonal anti-Syb2 (Synaptic Systems, 104211), rabbit polyclonal anti-Itsn1 (Millipore, ABS984) and anti-EndoA1 (see above)) and secondary antibodies (Li-COR IRDye 800 cw, goat anti-mouse IgG (H + L), 925-32210; Li-COR IRDye 680RD, goat anti-rabbit IgG (H + L), 925-68071) diluted in the blocking buffer at 1:1,000 for primaries and 1:10,000 for secondaries. The proteins were detected using the Odyssey infrared imaging system (Li-COR) and analyzed using Image Studio version 5.2 (a software package from Li-COR Biosciences) and/or ImageJ. Both software programs were used to compare the density (that is, intensity) of bands on a digital image of the western blot.

#### Immunoprecipitation

For Itsn1–Endo A1 protein-binding assessment, we homogenized whole mouse brains (~P60) in a homogenization buffer containing 0.32 M sucrose, 10 mM HEPES at 7.4 pH and Complete Protease Inhibitor (Roche, 04693132001). Homogenates were centrifuged and separated from supernatants. Lysis buffer containing 20 mM HEPES at pH 7.4, 50 mM KCl, 2 mM MgCl_2_ and 1% Triton X-100 was added to homogenates for 1 h with regular trituration on ice. To assess the Itsn1 rescue construct binding to Endo A1, *Itsn1* KO mouse hippocampal cultures were grown on 10 cm dishes (Corning, 353001) and infected with lentivirus containing rescue cassettes on DIV 7. On DIV 14, cells were lysed using the aforementioned buffer and triturated for 30 min on ice. Infected cells were compared to WT neurons and noninfected KO neurons from littermates. Cell or whole-brain lysates were spun down at 4 °C, and the supernatant was collected. Lysates were then bound to Dynabeads (Thermo Fisher Scientific, 10-001-D) per the manufacturer’s protocol, containing the abovementioned anti-endophilin antibody. Proteins were eluted from beads by heating in 2× SDS sample buffer diluted in water from a 4× stock (2 ml (1 M) Tris–HCl (pH 6.8), 0.8 g (10%) SDS, 2.4 ml 2-βMe, 4 ml glycerol, MilliQ up to 10 ml and bromophenol blue powder) at 70 °C for 10 min. Heated samples were then loaded into precast Tris–glycine 4–20% gradient gels (Bio-Rad, 4561095) and run at 140 V for 45 min. After, proteins were transferred to methanol-activated PVDF membranes (Bio-Rad, 1620177) by a wet transfer system at 200 V for 90 min. Membranes were blocked for 30 min at room temperature in intercept TBS blocking buffer (Li-COR, 927-60001) and then transferred into primary antibody solution (anti-endophilin 1 (1:1,000), anti-Itsn1 (Millipore; 1:1,000) and anti-β actin (Synaptic Systems, 251011; 1:5,000) in intercept TBS blocking buffer) overnight at 4 °C (shaking). Membranes were then washed thrice for 5 min in TBS-T and then transferred to a secondary antibody solution that contains IRDye secondary antibodies (Li-COR, as above or using Li-COR IRDye 800 cw, donkey anti-guinea pig IgG (H + L), 926-32411, where appropriate) diluted to 1:10,000 in intercept blocking buffer for 1 h at room temperature. Signal was detected using Li-COR Odyssey CLx (0958), and quantification was done by Image Studio version 5.2 from Li-COR.

#### iGluSnFR imaging and analysis

Neurons were removed from culture media and mounted in a Warner Instruments field stimulation chamber (RC-49MFSH). Neurons were continuously perfused with the physiological saline solution with 1.2 mM CaCl_2_ (as above), held at 37 °C by an in-line heater. Imaging was performed between DIV14 and DIV16 on a custom-built Zeiss AxioExaminer Z.1 equipped with a live-slice Yokogawa spinning-disk module, Flash4.0 V3 sCMOS camera and a ×40/1.0 NA water immersion objective. iGluSnFR fluorescence was excited by 488 nm laser light. Neurons transfected with iGluSnFR were stimulated for 1 ms at 60 V with a Grass Instruments SD9 Square Pulse Stimulator. Stimulation timing and intervals were programmed on an AMPI Master-8 Pulse Stimulator. Images were acquired at 7.5 ms (~133 Hz) exposure for 400 frames.

All images were background subtracted using a rolling ball radius of seven pixels (pixel size 0.645 μm × 0.645 μm). To discern between response and failure events, the average projection (created using the prestimulus fluorescence baseline) was subtracted from the maximum projection (created using the entire image series). ROIs were then manually selected with a 1-click ROI around puncta. All ROIs were manually examined for failure to respond or aberrant signal profiles, and in these cases, ROIs were omitted. The iGluSnFR fluorescence measurement was expressed as (*F*(*t*) − *F*0)/*F*0 = d*F*/*F*0, where *F*(*t*) is fluorescence intensity over time, and *F*0 is the baseline intensity averaged over ~300 ms before the first stimulus. Peak *dF*/*F* values from responding synapses were then extrapolated from d*F*/*F* traces by automated segmentation with manual quality control of peak fitting. Paired-pulse experiments were done sequentially in the same cell (50 ms to 1 s). To quantify PPR values, we divided the second *dF*/*F* peak from the initial peak across all paired-pulse time intervals. For all PPRs, values >30 and <0 were omitted. To account for increased failure rates in the initial stimulation by the 1-s experiment and expected return to baseline (PPR ~1), PPRs >5 and <0 were omitted. Sample sizes were determined as in experiments conducted previously^21^.

#### Electrophysiology and data analysis

For *Itsn1* KO experiments, adult *Itsn1* WT and KO mice of both sexes, ranging from 6 to 8 weeks of age, were anaesthetized using a combination of isoflurane inhalation and avertin injection. Mice underwent cardiac perfusion using chilled sucrose solution (10 mM NaCl, 2.5 mM KCl, 10 mM glucose, 84 mM NaHCO_3_, 120 mM NaH_2_PO_4_, 195 mM sucrose, 1 mM CaCl_2_ and 2 mM MgCl_2_) saturated with 5% oxygen/95% carbon dioxide (carbogen). Brain was rapidly dissected, and the hippocampi were removed. Hippocampi were then embedded in premade agarose molds and sliced at 400 µm using a Leica VT1200S vibratome at a speed of 0.05 mm s^−1^ and an amplitude of 1.0 mm. Slices were then transferred to artificial cerebrospinal fluid (ACSF; 119 mM NaCl, 2.5 mM KCl, 1.3 mM MgSO_4_, 2.5 mM CaCl_2_, 26 mM NaHCO_3_, 1 mM NaH_2_PO_4_ and 11 mM d-glucose (315 Osm, pH 7.4)) heated using a water bath to 32 °C saturated with carbogen. Slices were recovered at this temperature for 15 min before being removed from the bath and recovered for 1 h at room temperature.

Recordings were performed at 32 °C in ACSF. Glass pipettes containing silver chloride electrodes were used to both stimulate and record. The stimulating electrode was filled with ACSF and placed in CA1, while the recording electrode was filled with 1 M NaCl and placed to record from CA1/CA2 synapses in CA2. To record paired-pulse measurements, a bipolar square pulse of 0.3 ms at 60 mV was applied, followed by a second pulse at varying intervals ranging from 20 to 1,000 ms. To assess release probability, we acquired data from a range of stimulation strengths, starting with 20 mV and increasing in intervals of 20–100 mV, which revealed a linear relationship between fEPSP slope and fiber volley amplitude. The slope and *y*-intercept of this line were used to identify relative differences in release probability.

To assess overall release, we applied a depressing train stimulation consisting of 100 bipolar square pulses (0.3 ms duration, 60 mV amplitude) delivered at 20 Hz. To assess synaptic recovery after train stimulation, a single pulse was applied at varying intervals following the end of the train (100 pulses, 20 Hz) at various intervals ranging from 100 ms to 3 s. Recordings were taken using Multiclamp 700B and Digidata 1550B and Clampex v11.2 software. Stimulus was applied using the A-M Systems Isolated Pulse Stimulator Model 2100. Traces were analyzed using a combination of Clampfit software v11.2 and custom MATLAB code gifted by the E. Chapman Lab.

#### Statistical analysis

Detailed statistical information is collated in Supplementary Table 1.

Internal FRAP experiments yielded Tau measurements that were compared using a Student’s *t*-test, as Tau values were normally distributed. An *α* of 0.05 was set for null hypothesis testing. Different HEK293T cell cultures (*n*) were imaged on separate days. Condensates that were assayed (*n*) were taken from multiple cells within a culture.

Molecular condensates (*n*) were quantified from three to four biological replicates per condition. Each biological replicate was a separate HEK293 culture (*n*). Multiple comparisons were made by either a Kruskal–Wallis test or one-way analysis of variance (ANOVA), depending on normality of distributions. For Kruskal–Wallis tests, each condition was compared by Dunn’s multiple comparisons test. An *α* of 0.05 was set for null hypothesis testing.

2D STED images were acquired from two to three biological replicates per condition. Each replicate was a dissociated mouse hippocampal culture (*n*) taken from different mice on different days. For each *n*, roughly 50–100 presynaptic bouton ROIs (*n*) were imaged from multiple cells. ROIs from each replicate were pooled and quantified as previously described^36^. An α of 0.05 was set for null hypothesis testing. For Itsn1 and EndoA1 foci distance statistical analysis, pooled distance measurements from each condition were assessed for distribution normality. A full pairwise Kruskal–Wallis test was performed. Afterward, each condition was compared by Dunn’s multiple comparisons test.

CV analysis was performed from three biological replicates. Each replicate was a dissociated mouse hippocampal culture (*n*). Multiple axons were measured per replicate (*n*). An *α* of 0.05 was set for null hypothesis testing. Calculated CVs were compared by paired Student’s *t*-tests.

For electron microscopy data, measurements were taken from roughly 100 synaptic profile micrographs (*n*) per condition. Replicate high-pressure freezing experiments (*n*) were conducted with cultures taken from different mice on different days. Sample sizes for each replicate were inferred from previous flash-and-freeze experiments as opposed to power analysis. An *α* of 0.05 was set for null hypothesis testing. For count datasets such as these, non-normal, nonparametric distributions are typically assumed. However, means are best to represent central tendency, and these data are binomially distributed. So, an ANOVA test with a Brown–Forsythe correction and Games–Howell post hoc was conducted. In the case of electron microscopy datasets with measurements of 0, Brown–Forsythe correction fails. Therefore, a statistical comparison was performed using a Kruskal–Wallis test followed by Dunn’s multiple comparisons. In cases where only two samples were compared, a Student’s *t*-test with Brown–Forsythe correction and Games–Howell post hoc was conducted. Datasets that contained measurements of 0 were instead compared using a Mann–Whitney test.

For iGluSnFR data, d*F*/*F* traces were taken from 20 to 100 release sites (*n*) from ~8 cells taken from two to three different cultures (*n*) for both *Itsn1* WT and *Itsn1* KO. An *α* of 0.05 was set for null hypothesis testing. Second peak *dF*/*F* measurements and PPRs from each genotype were assessed for distribution normality. A full pairwise Kruskal–Wallis test was then performed. Afterward, each condition was compared by Dunn’s multiple comparisons test. For the first peak d*F*/*F* measurements, values were assessed for distribution normality. Then, values were compared by a Mann–Whitney test.

For electrophysiological data, fEPSP measurements were taken three times per experimental condition, per hippocampal slice (*n*). Approximately two to five slices were taken from each animal (*n*), approximately six of which were measured per genotype, as reported previously^59^. An *α* of 0.05 was set for null hypothesis testing. To compare the average amplitude of the first ten and last ten stimulations, fEPSP amplitudes taken from 100 AP, 20 Hz trains in either *Itsn1* WT or KO slices were pooled and compared by an unpaired Student’s *t*-test. For linear back-extrapolation analysis, normalized cumulative peak amplitudes were taken from 100 AP, 20 Hz trains conducted on either *Itsn1* WT or KO slices and back extrapolated by linear regression analysis. The *y* intercepts were pooled and compared between *Itsn1* WT and KO measurements by an unpaired Student’s *t*-test.

Data were randomized and blinded wherever feasible. All data met the assumptions of the statistical tests used. Where tested, normality and equal variances statements are reported in Supplementary Table 1. If not tested, data distributions were assumed, and data distributions are shown in Supplementary Table 1 and source data. All statistical analyses were conducted in GraphPad Prism.

### Reporting summary

Further information on research design is available in the Nature Portfolio Reporting Summary linked to this article.

## Online content

Any methods, additional references, Nature Portfolio reporting summaries, source data, extended data, supplementary information, acknowledgements, peer review information; details of author contributions and competing interests; and statements of data and code availability are available at 10.1038/s41593-025-02002-4.

## Supplementary information

Reporting Summary

Supplementary Table 1 Statistical data for Figs. 1–7 and Extended Data Figs. 1, 2, 5–8 and 10.

## Source data

Source Data Fig. 1 Statistical source data.

Source Data Fig. 2 Statistical source data.

Source Data Fig. 2 Blot 1: Unprocessed western blot of clathrin HC protein in synaptic vesicle and clathrin-coated vesicle isolates. Blot 2: Unprocessed western blot of endophilin A1 protein in synaptic vesicle and clathrin-coated vesicle isolates. Blot 3: Unprocessed western blot of ITSN1 protein in synaptic vesicle and clathrin-coated vesicle isolates. Blot 4: Unprocessed western blot of synaptophysin and VAMP2 protein in synaptic vesicle and clathrin-coated vesicle isolates.

Source Data Fig. 3 Statistical source data.

Source Data Fig. 4 Statistical source data.

Source Data Fig. 5 Statistical source data.

Source Data Fig. 6 Statistical source data.

Source Data Fig. 7 Statistical source data.

Source Data Extended Data Fig.1 Statistical source data.

Source Data Extended Data Fig. 2 Statistical source data.

Source Data Extended Data Fig. 3 Statistical source data.

Source Data Extended Data Fig. 4 Statistical source data.

Source Data Extended Data Fig. 5 Statistical source data.

Source Data Extended Data Fig. 5 Blot 1: Unprocessed western blot of intersectin-1 and actin protein in input lysates to the left of endophilin A1 pulldowns conducted in WT, KO, KO^+^ITSN1 WT and KO^+^ITSN1 WEYE lysates, in that order (left to right). Blot 2: Unprocessed western blot of endophilin A1 protein in input lysates to the left of endophilin A1 pulldowns conducted in WT, KO, KO^+^ITSN1 WT and KO^+^ITSN1 WEYE lysates, in that order (left to right).

Source Data Extended Data Fig. 6 Statistical source data.

Source Data Extended Data Fig. 7 Statistical source data.

Source Data Extended Data Fig. 8 Statistical source data.

Source Data Extended Data Fig. 9 Statistical source data.

Source Data Extended Data Fig. 10 Statistical source data.

## Data Availability

Original images used in this work will be uploaded to figshare. Additional data and details are provided in Source Data. Data will be available upon request. Source data are provided with this paper.

## Code availability

Custom R, MATLAB and Fiji scripts for electron microscopy analysis are available at https://github.com/shigekiwatanabe/SynapsEM. STED analysis codes are available at https://github.com/imotolab-neuroem/STED_image_analysis_package_public_v1.4/tree/main. iGluSnFR MATLAB analysis scripts are available upon request. Electrophysiology analysis codes are available upon request.
