# Supplementary material for: Intersectin and endophilin condensates prime synaptic vesicles for release site replenishment
Source: Nat Neurosci. 2025 Jul 8;28(8):1649–62. doi: 10.1038/s41593-025-02002-4 (PMC12321584; doi:10.1038/s41593-025-02002-4)
Supplement: Supplementary file 1 — Reporting Summary [file 41593_2025_2002_MOESM1_ESM.pdf]

nature portfolio

Corresponding author(s): Dr. Shigeki Watanabe

Last updated by author(s): 4/8/2025

Reporting Summary

Nature Portfolio wishes to improve the reproducibility of the work that we publish. This form provides structure for consistency and transparency in reporting. For further information on Nature Portfolio policies, see our Editorial Policies and the Editorial Policy Checklist.

Statistics

For all statistical analyses, confirm that the following items are present in the figure legend, table legend, main text, or Methods section.

n/a	Confirmed	
☐	☒	The exact sample size (*n*) for each experimental group/condition, given as a discrete number and unit of measurement
☐	☒	A statement on whether measurements were taken from distinct samples or whether the same sample was measured repeatedly
☐	☒	The statistical test(s) used AND whether they are one- or two-sided<br>*Only common tests should be described solely by name; describe more complex techniques in the Methods section.*
☐	☒	A description of all covariates tested
☐	☒	A description of any assumptions or corrections, such as tests of normality and adjustment for multiple comparisons
☐	☒	A full description of the statistical parameters including central tendency (e.g. means) or other basic estimates (e.g. regression coefficient) AND variation (e.g. standard deviation) or associated estimates of uncertainty (e.g. confidence intervals)
☐	☒	For null hypothesis testing, the test statistic (e.g. *F*, *t*, *r*) with confidence intervals, effect sizes, degrees of freedom and *P* value noted<br>*Give P values as exact values whenever suitable.*
☒	☐	For Bayesian analysis, information on the choice of priors and Markov chain Monte Carlo settings
☒	☐	For hierarchical and complex designs, identification of the appropriate level for tests and full reporting of outcomes
☒	☐	Estimates of effect sizes (e.g. Cohen's *d*, Pearson's *r*), indicating how they were calculated

Our web collection on statistics for biologists contains articles on many of the points above.

Software and code

Policy information about availability of computer code

Data collection	AMT Capture v6 for electron microscopy data acquisition. Zen blue and black for Airyscan imaging, confocal imaging, and iGluSnFR imaging. Imspector software v16.3 for 3-color 2D STED imaging. Clampex/Clampfit 11.2 software for electrophysiology. Li-COR software package for western blot imaging.
Data analysis	Imspector software v16.3 for STED analysis. SynapsEM analysis codes for electron microscopy analysis, Custom analysis scripts for Matlab (v2015 or newer). GraphPad Prism 7 or newer, ImageJ, Image Studio v5.2. R Studio + R for Windows v4.41 for data randomization.

For manuscripts utilizing custom algorithms or software that are central to the research but not yet described in published literature, software must be made available to editors and reviewers. We strongly encourage code deposition in a community repository (e.g. GitHub). See the Nature Portfolio guidelines for submitting code & software for further information.

Data

Policy information about availability of data

All manuscripts must include a data availability statement. This statement should provide the following information, where applicable:

- Accession codes, unique identifiers, or web links for publicly available datasets
- A description of any restrictions on data availability
- For clinical datasets or third party data, please ensure that the statement adheres to our policy

Source data will be published with the manuscript. Images are available upon request. Matlab scripts are posted on Github but also available upon request.

Research involving human participants, their data, or biological material

Policy information about studies with human participants or human data. See also policy information about sex, gender (identity/presentation), and sexual orientation and race, ethnicity and racism.

Reporting on sex and gender	N/A
Reporting on race, ethnicity, or other socially relevant groupings	N/A
Population characteristics	N/A
Recruitment	N/A
Ethics oversight	N/A

Note that full information on the approval of the study protocol must also be provided in the manuscript.

Field-specific reporting

Please select the one below that is the best fit for your research. If you are not sure, read the appropriate sections before making your selection.

☒ Life sciences ☐ Behavioural & social sciences ☐ Ecological, evolutionary & environmental sciences

For a reference copy of the document with all sections, see nature.com/documents/nr-reporting-summary-flat.pdf

Life sciences study design

All studies must disclose on these points even when the disclosure is negative.

Sample size	Power analysis was not used to determine the number (n) of synaptic profiles (2D EM). Our threshold of n >200 (from N = 2 or more experiments) for synaptic profiles was taken from previous work (Watanabe et al., 2013), based on 15-20% of synapses containing endocytic or exocytic events, such that >30 synapses with endocytic or exocytic events would be captured. For 2D STED fluorescence imaging, n>30 ROI's (from N=2 independent cultures) were chosen for each experiment. For live imaging, n>300 ROI's (from N=3 independent cultures) were chosen for each experiment. For molecular condensate analysis, multiple cells (n) were imaged from 3-4 (N) separate cultures. For electrophysiology data, 2-5 hippocampal slices (n) were taken from ~6 animals (N). For iGluSnFR data, multiple synapses (n) from multiple cells were imaged from 3-4 (N) separate cultures.
Data exclusions	Images that could not be reliably segmented, either because the image was not of a bona fide synapse or morphology was too poor, were excluded from segmentation; this was done only after randomizing the images. Technical replicate of one zap-and-freeze condition of uncertain identity; stimulation type could not be verified. Electrophysiology sweeps in which noise made data analysis intractable. iGluSnFR synapses which displayed noise masking true signal. No other data were excluded.
Replication	All experiments were performed at least twice (from separate litters, different rounds of neuronal cell/HEK293T cell culture, frozen and processed separately, and segmented in separate batches of randomized images). Similar results were obtained in each experiment.
Randomization	No randomization into experimental groups was performed prior to freezing. For image segmentations, images were always randomized before manual segmentation.
Blinding	To limit bias, synapses were found by bidirectional raster scanning along the section at 100,000x, which makes it difficult to “pick” certain synapses, as a synapse usually takes up most of this field of view, and anything that appeared to be a synapse was imaged without close examination. For electrophysiology, animal genotypes were blinded during before and during experiments.

Reporting for specific materials, systems and methods

We require information from authors about some types of materials, experimental systems and methods used in many studies. Here, indicate whether each material, system or method listed is relevant to your study. If you are not sure if a list item applies to your research, read the appropriate section before selecting a response.

Materials & experimental systems

n/a	Involved in the study
	☒ Antibodies
	☒ Eukaryotic cell lines
☒	☐ Palaeontology and archaeology
	☒ Animals and other organisms
☒	☐ Clinical data
☒	☐ Dual use research of concern
☒	☐ Plants

Methods

n/a	Involved in the study
☒	☐ ChIP-seq
☒	☐ Flow cytometry
☒	☐ MRI-based neuroimaging

Antibodies

Antibodies used

Primary Antibodies (clone number stated where possible): Anti-Bassoon Mouse Monoclonal Enzo Life Sciences Cat no SAP7F407, Anti-Bassoon Mouse Monoclonal Synaptic Systems Cat no 141 011, Anti-Endophilin 1 Guinea Pig Polyclonal Synaptic Systems Cat no 159 004, Anti-Intersectin-1 Rabbit Polyclonal (Gift from Volker Haucke Lab), Anti-Synapsin 1/2 Guinea Pig Polyclonal Synaptic Systems Cat no 106 004, Anti-Synaptobrevin 2 Mouse Monoclonal Synaptic Systems Cat no 104 211, Anti-GFP Rabbit Polyclonal MBL International, Cat no 598, Anti-Rim1 Mouse Monoclonal Synaptic Systems Cat no 140 111, Anti-Intersectin-1 Rabbit Polyclonal Millipore Cat no ABS984, Anti-Beta Actin Mouse Monoclonal Synaptic Systems Cat no 251 011, Anti-Clathrin HC Mouse Monoclonal Abcam Cat no Ab2731, Anti-Synaptophysin-1 Mouse Monoclonal Synaptic Systems Cat no 101011
Secondary Antibodies: Anti-Rabbit Atto647N Rockland 611-156-122S, Anti-Mouse Atto647N Rockland 610-156-121S, Anti-Mouse/Rabbit/Guinea Pig Alexa594 Invitrogen A11005, A11012, A11076, Anti-Mouse STAR 460L Aberrior ST460L-1001, Li-COR IRDye 800 cw, goat anti-mouse IgG (H + L), 925-32210; Li-COR IRDye 680RD, goat anti-rabbit IgG (H+L) 925-68071, Li-COR IRDye 800 cw, donkey anti-guinea pig IgG (H + L), 926-32411
Nanobodies: anti-PSD-95 Fluotag-2x nanobody Atto643 NanoTag Biotechnologies N3702-At643-L

Validation

Anti-Bassoon Enzo: https://www.enzo.com/product/bassoon-monoclonal-antibody-sap7f407/
Anti-Bassoon SySy: https://sysy.com/product/141011
Anti-Endophilin 1: https://sysy.com/product/159004
Anti-Intersectin-1 (Gift from Volker Haucke Lab): used as reported in: https://doi.org/10.1016/j.celrep.2019.12.035
Anti-Synapsin 1/2: https://sysy.com/product/106004 (K.O. validated)
Anti-Synaptobrevin 2: https://sysy.com/product/104211 (K.O validated)
Anti-GFP: https://www.mblintl.com/products/598/
Anti-Intersectin-1 (Millipore): https://www.emdmillipore.com/US/en/product/Anti-Intersectin-1-Antibody,MM_NF-ABS984
Anti-Beta Actin: https://sysy.com/product/251011
Anti-Clathrin HC: https://www.abcam.com/en-us/products/primary-antibodies/clathrin-heavy-chain-antibody-x22-ab2731?srsltid=AfmBOorSqtUl7gXRvjnRHjzFRWdo_gpQQU3cALoJj7fetxPlNiE8dopZ
Anti-Synaptophysin-1: https://www.sysy.com/product/101011
Anti-PSD-95 Nanobody: https://nano-tag.com/product/fluotag-x2-anti-psd95/

Eukaryotic cell lines

Policy information about cell lines and Sex and Gender in Research

Cell line source(s)	HEK293T (ATCC, CRL-3216) , HEK293 (ATCC, CRL-1573) cell lines--human embryonic kidneys.
Authentication	None of the cell lines used were authenticated.
Mycoplasma contamination	Cell lines were not tested for Mycoplasma contamination.
Commonly misidentified lines (See ICLAC register)	N/A

Animals and other research organisms

Policy information about studies involving animals; ARRIVE guidelines recommended for reporting animal research, and Sex and Gender in Research

Laboratory animals	E18 or P0 wild-type C57BL/6J, EndoA TKO C57BL/6J and 129SV/JItsn1- mice of both sexes were used for all culture experiments. The sex of newborn or embryonic pups cannot be identified, but cells for neuronal culture were pooled from all the mice in a litter, and so contained cells from mice of both sexes in each experiment. Whole brain and slices were taken from male and female animals 6-8 weeks of age.
Wild animals	None used.
Reporting on sex	N/A
Field-collected samples	None.

Ethics oversight	Animal care was performed according to the National Institutes of Health guidelines for animal research with approval from the Animal Care and Use Committee at the Johns Hopkins University School of Medicine, or complied with the national animal care guidelines and were approved by the University Medical Center Göttingen board for animal welfare and the animal welfare office of the state of Lower Saxony (LAVES)

Note that full information on the approval of the study protocol must also be provided in the manuscript.

Plants

Seed stocks	N/A
Novel plant genotypes	N/A
Authentication	N/A
