# Supplementary figures and images for: Intersectin and endophilin condensates prime synaptic vesicles for release site replenishment
Source: Nat Neurosci. 2025 Jul 8;28(8):1649–62. doi: 10.1038/s41593-025-02002-4 (PMC12321584; doi:10.1038/s41593-025-02002-4)

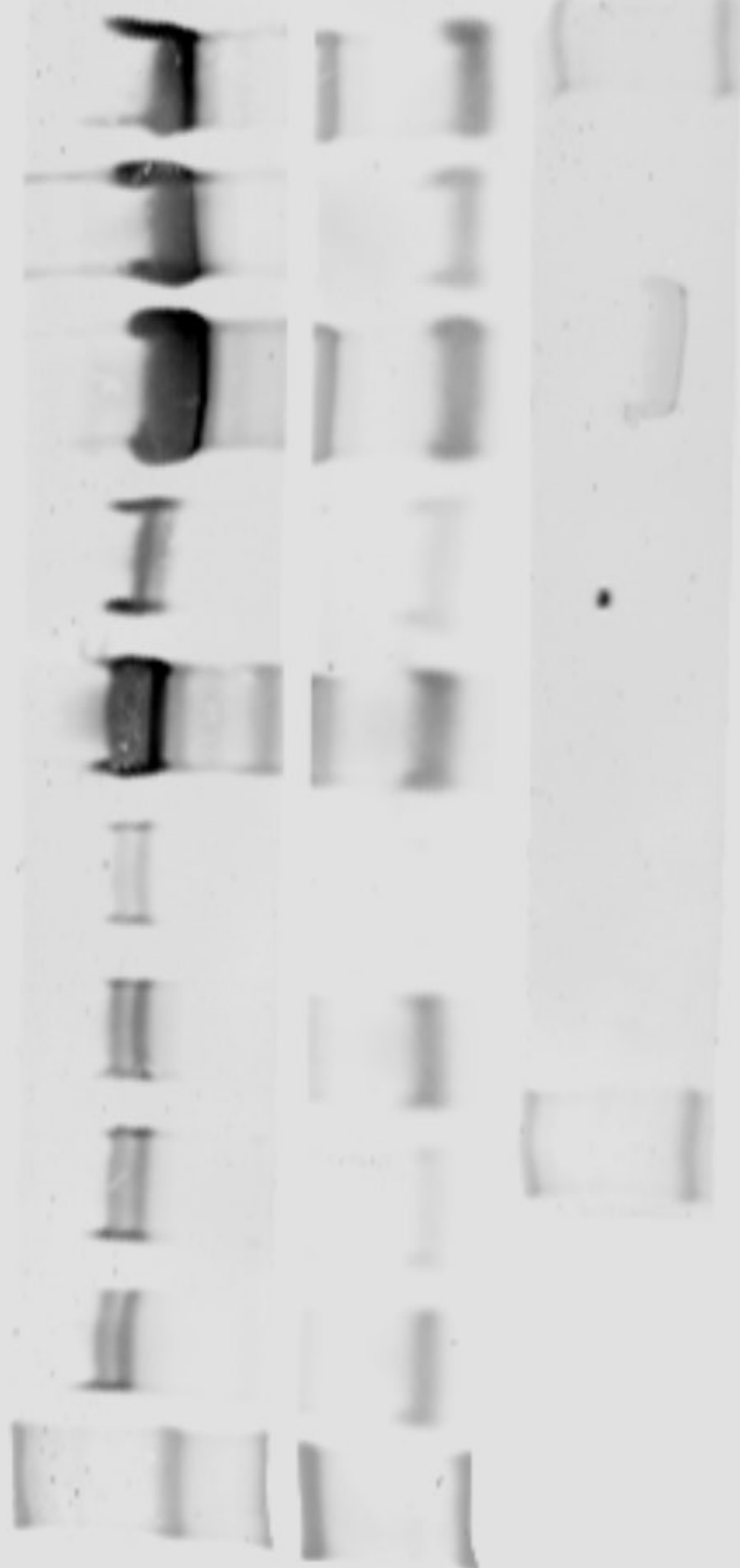

111 50 36 56

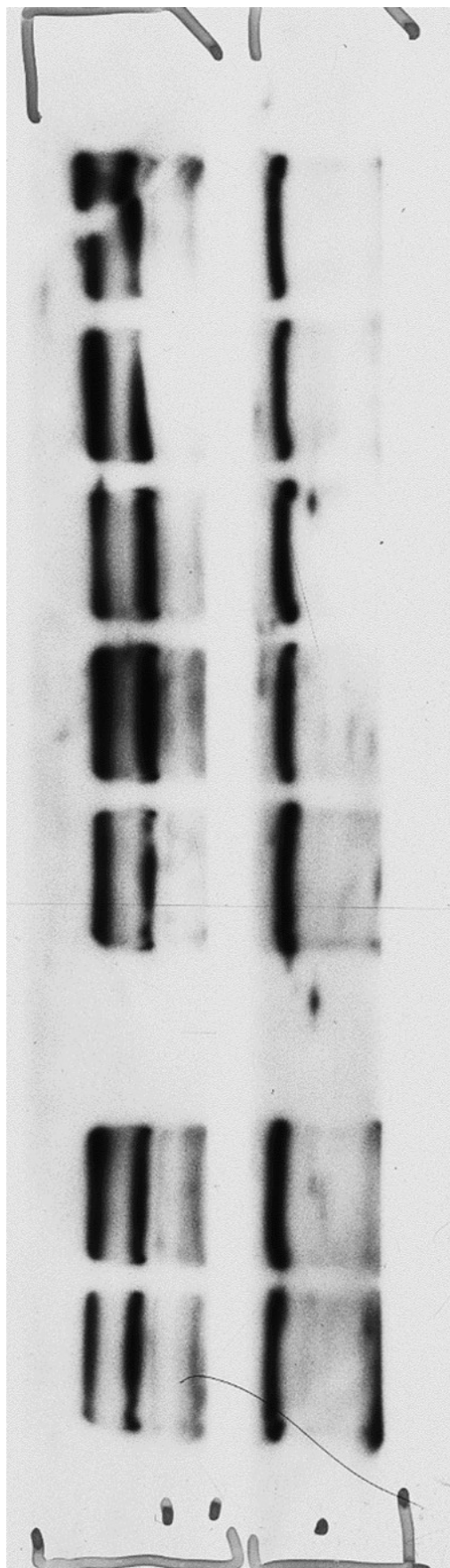

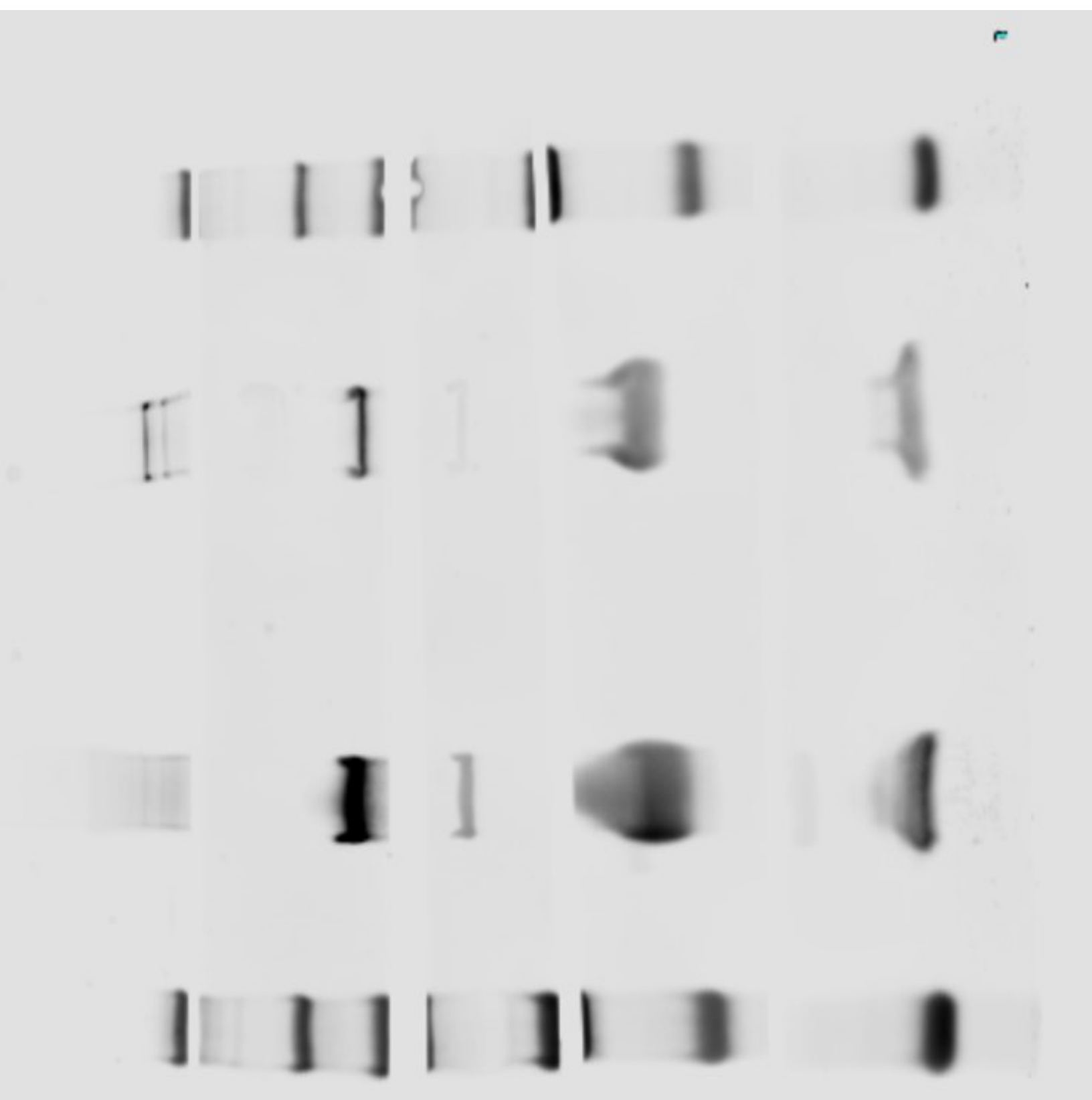

Supplement: Supplementary file 5 — Blot 1: Unprocessed western blot of clathrin HC protein in synaptic vesicle and clathrin-coated vesicle isolates. Blot 2: Unprocessed western blot of endophilin A1 protein in synaptic vesicle and clathrin-coated vesicle isolates. Blot 3: Unprocessed western blot of ITSN1 protein in synaptic vesicle and clathrin-coated vesicle isolates. Blot 4: Unprocessed western blot of synaptophysin and VAMP2 protein in synaptic vesicle and clathrin-coated vesicle isolates. [file 41593_2025_2002_MOESM5_ESM.pdf]

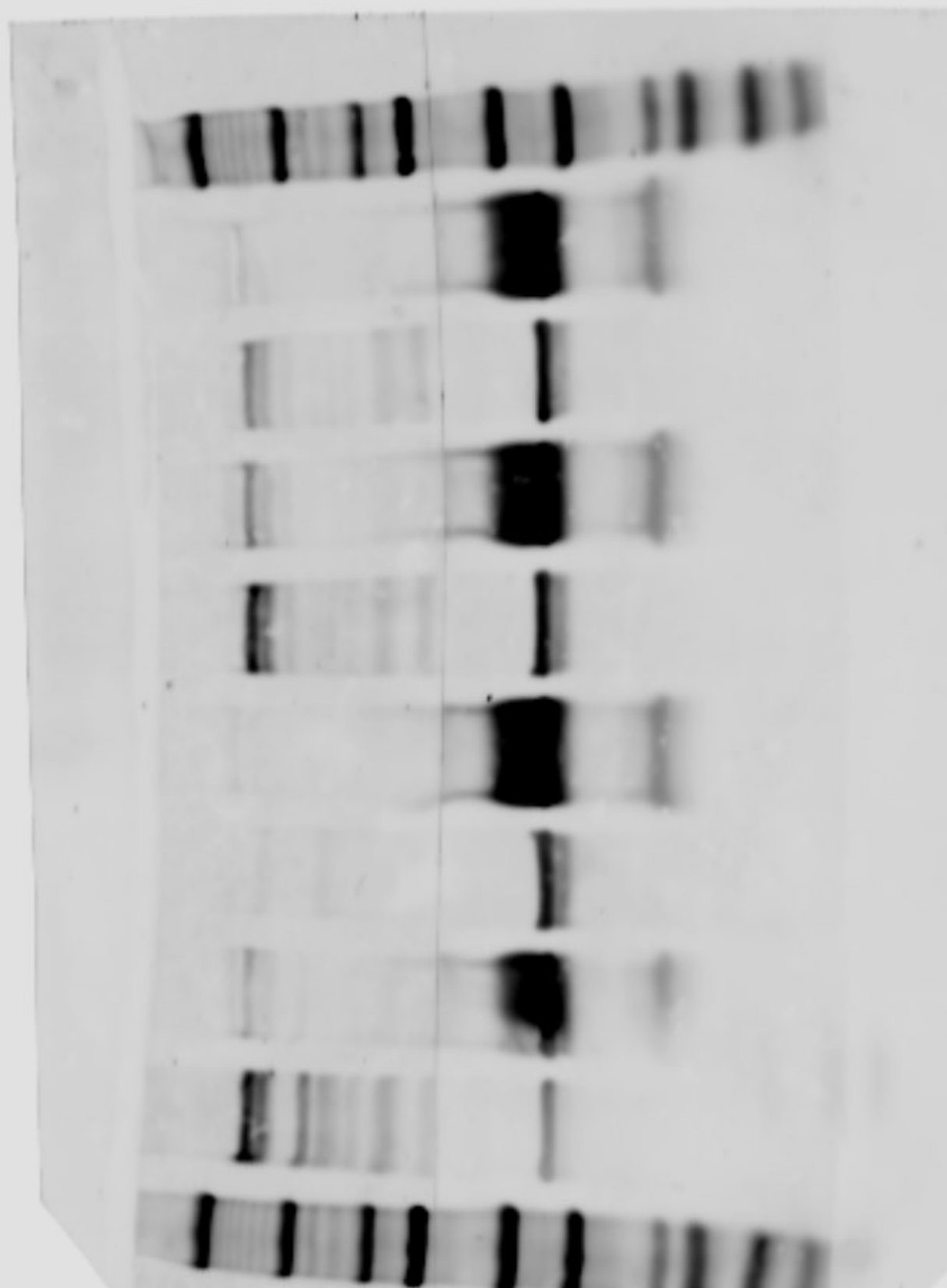

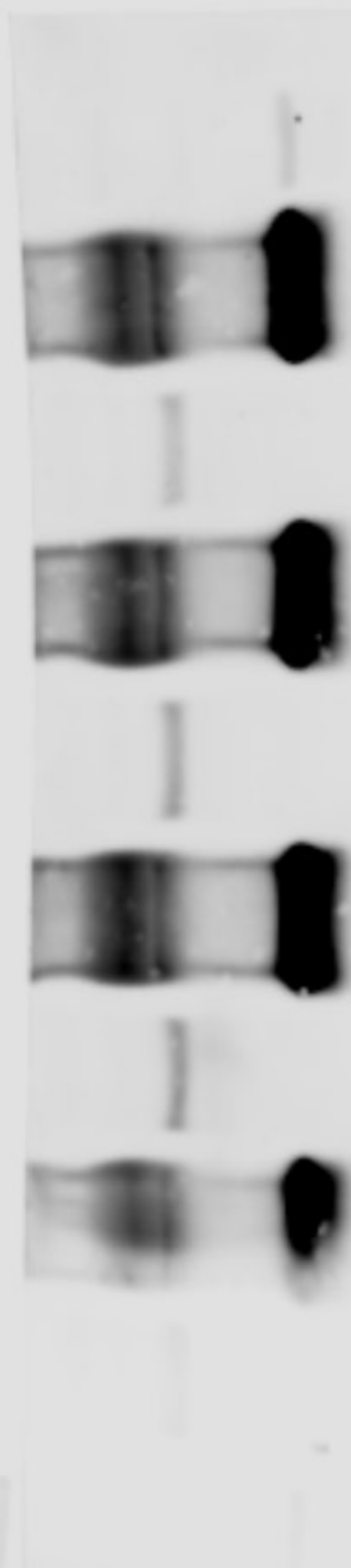

Supplement: Supplementary file 16 — Blot 1: Unprocessed western blot of intersectin-1 and actin protein in input lysates to the left of endophilin A1 pulldowns conducted in WT, KO, KO+ITSN1 WT and KO+ITSN1 WEYE lysates, in that order (left to right). Blot 2: Unprocessed western blot of endophilin A1 protein in input lysates to the left of endophilin A1 pulldowns conducted in WT, KO, KO+ITSN1 WT and KO+ITSN1 WEYE lysates, in that order (left to right). [file 41593_2025_2002_MOESM16_ESM.pdf]
